# Effect of a wearable-based individualised multidomain lifestyle intervention on cognitive and physical function in older adults at risk of decline (INSPIOR): a single-centre, open-label, randomised controlled trial

**DOI:** 10.1016/j.eclinm.2026.104212

**Published:** 2026-09-23

**Authors:** Keisuke Sakurai, Ryo Tanaka, Joseph Ledsam, Izumi Shiraishi, Hiroyasu Kasahara, Saya Anzai, Miwa Watanabe, Momoko Funakawa, Saori Shimada, Tatsuya Goto, Riki Kawabata, Keiko Murasakino, Tomoko Kawaura, Noriko Inamura, Ko Kaneta, Satoru Kobayashi, Toshiyuki Nomura, Nori Karasawa, Takashi Matsumoto, Tomoki Taira, Toshihiro Ishiguro, Kazuyuki Kudo, Matthew J. Thompson, Hiroki Kayama, Yukihiro Sugawara, Shin'ichi Warisawa, Tatsuhiro Hisatsune

**Affiliations:** a Department of Integrated Biosciences, Graduate School of Frontier Sciences, The University of Tokyo, Kashiwa, Japan; b Google, Mountain View, CA, USA; c Urban Design Center Kashiwanoha (UDCK), Kashiwa, Japan; d Community Health Promotion Laboratory, Mitsui Fudosan, Kashiwa, Japan; e Planning Group, Kashiwanoha Urban Planning and Development Department, Mitsui Fudosan, Kashiwa, Japan; f Research and Development Center, NH Foods, Tsukuba, Japan; g Technology Development Office, NH Foods, Joso, Japan; h Customer Service Department, NH Foods, Shinagawa, Japan; i Department of Human and Engineered Environmental Studies, Graduate School of Frontier Sciences, The University of Tokyo, Kashiwa, Japan; j AI Symbiotic Future Society Program, Graduate School of Arts and Sciences, The University of Tokyo, Tokyo, Japan; k Smart Health Initiative, Graduate School of Frontier Sciences, The University of Tokyo, Kashiwa, Japan

**Keywords:** Multidomain lifestyle intervention, Wearable device, Cognitive function, Cognitive ageing, Older adults

## Abstract

**Background:**

Multidomain lifestyle programmes are the leading preventive approach to cognitive decline, but they rely on intensive face-to-face delivery that is hard to scale. We assessed whether an automated, wearable-based multidomain programme of activity, sleep, and optional nutrition prompts changed cognitive and physical function in at-risk older adults.

**Methods:**

INSPIOR was a 6-month, single-centre, open-label, randomised controlled trial in Kashiwa, Japan. Adults aged 65–92 years with low body-mass index, pre-frailty, frailty, or sarcopenia-related decline were randomly assigned 1:1, in permuted blocks of four and without stratification, to the Fitbit-based intervention or an assessment-only control; allocation was not concealed and outcome assessors were not masked. Co-primary outcomes were the Montreal Cognitive Assessment (MoCA), Trail Making Test (TMT), and skeletal muscle mass index (SMI), with no pre-specified hierarchy or multiplicity adjustment; analyses used intention-to-treat analysis of covariance with multiple imputation. This trial was registered with UMIN-CTR, UMIN000052707, and ClinicalTrials.gov, NCT06135740.

**Findings:**

Between Nov 8, 2023, and Jul 31, 2024, 355 participants were randomly assigned to the intervention group (n = 178) or control group (n = 177); all were analysed. Adjusted between-group differences were 0·58 MoCA points (95% CI 0·03–1·13; p = 0·039; Holm-adjusted p = 0·12), 0·16 s (−5·07 to 5·40; p = 0·95) for TMT-A, 5·59 s (−3·88 to 15·05; p = 0·25) for TMT-B, and −0·01 kg/m^2^ (−0·09 to 0·07; p = 0·88) for SMI. Mild skin irritation and dietary-recording stress occurred; no serious adverse event was intervention-related.

**Interpretation:**

The intervention was associated with a small difference in a global cognitive screening score that did not survive multiplicity adjustment and is of uncertain clinical significance; the other two showed none. Larger, longer, masked trials are needed.

**Funding:**

University of Tokyo–NH Foods Collaborative Research Programme, 10.13039/100006785Google, Mitsui Fudosan.

**Research in context**

**Evidence before this study**

The trial was designed in 2023, before it was registered in November of that year. At that time the evidence for multidomain lifestyle prevention of cognitive decline rested mainly on the FINGER trial, which showed that a 2-year programme of diet, exercise, cognitive training, and vascular risk monitoring slowed cognitive decline in at-risk older adults, and on a Cochrane review of multidomain interventions, which found small and inconsistent cognitive benefits and no clear effect on dementia incidence; the trials in that review were heterogeneous in population, components, and outcome measures, and all delivered their interventions through intensive face-to-face or human-led coaching. Randomised trials of consumer wearables and mobile-health applications had shown that automated prompting can increase physical activity in older adults, but these were mostly small, unmasked, and short, and rarely measured cognition. Evidence for the nutritional component was the weakest of the three: an umbrella review of prospective studies graded most individual nutrients as low or very-low quality, and a randomised trial of the MIND diet showed no benefit for global cognition over an active control diet. No pilot study or preliminary data for this intervention existed; the design rested entirely on published evidence. For this report we searched PubMed and Embase from database inception to Jan 5, 2026, without language restriction, using combinations of the terms “multidomain lifestyle intervention”, “wearable”, “physical activity”, “sleep”, “nutrition”, and “cognitive function” or “dementia”; we considered randomised trials of multidomain or wearable-delivered lifestyle interventions in older adults, and systematic reviews of such trials. We found no completed randomised trial of a wearable-based multidomain intervention in which the behavioural content was generated and delivered automatically from objective daily lifelog data, with a cognitive outcome among the primary outcomes. U.S. POINTER, Maintain Your Brain, J-MINT, and SMARRT have reported since this trial began, with mixed primary cognitive results; none evaluates such a programme.

**Added value of this study**

INSPIOR is, to our knowledge, the first randomised clinical trial of a wearable-based multidomain intervention whose behavioural content was generated and delivered without human involvement, in a risk-enriched community-dwelling older population using objective daily behavioural data. It shows that such a programme can be run with human contact limited to device setup and technical support, and that the difference it produced on a widely used cognitive screening measure was small and of uncertain clinical significance. It also shows what this design cannot settle: the trial was open-label with unmasked outcome assessment, three outcomes were registered as co-primary without a hierarchy or a multiplicity strategy, and the exploratory analyses that follow the primary comparison were all built on the one outcome that showed a difference.

**Implications of all the available evidence**

Wearable-centric multidomain programmes with automated prompt delivery may offer a scalable route to supporting healthy ageing in community settings, but this trial does not establish that they prevent cognitive decline. The behavioural changes observed—particularly in physical activity—are correlates of engagement rather than established pathways, and larger and longer trials with masked outcome assessment are needed to determine whether an automated programme yields a cognitive benefit that is durable and clinically meaningful.

## Introduction

Preserving cognitive function and delaying dementia are urgent public-health priorities as populations age.^1^ Multidomain interventions targeting several modifiable risk factors (vascular risk, physical inactivity, poor nutritional status, and limited cognitive reserve) have been the dominant preventive strategy, first demonstrated in the FINGER trial^2^ and tested subsequently in pragmatic trials and culturally adapted analogues.^3^, ^4^, ^5^ Results have varied, and a Cochrane synthesis^6^ concluded that they confer small but inconsistent cognitive benefits. Broad implementation remains difficult when programmes depend on frequent face-to-face coaching.

Wearable devices and smartphone applications can deliver such programmes without continuous professional input; consumer activity trackers increase daily step counts,^7^ and mobile-health interventions tailored to older people are useable and modestly effective at promoting physical activity.^8^

Indicators of physical vulnerability (eg, low body-mass index, pre-frailty or frailty, and sarcopenia-related decline) do not appear among the 14 modifiable dementia risk factors identified by the Lancet Commission.^1^ Physical frailty has nonetheless been associated with a higher incidence of cognitive disorders in community-dwelling older adults,^9^ so such populations are enriched for cognitive as well as physical events.

Higher daily step counts^10^ and greater volumes of structured exercise^11^ are associated with lower dementia incidence, and both short and long sleep duration are associated with higher long-term dementia risk.^12^ The evidence for nutrition is weaker: an umbrella review graded the evidence for most individual nutrients as low or very low,^13^ and a randomised trial of the MIND diet did not improve global cognition relative to an active control diet.^14^

INSPIOR tested whether a wearable-based, individualised multidomain intervention delivering automated behavioural prompts could improve cognitive and physical function in older adults at increased risk of decline, compared with an assessment-only control.

## Methods

### Study design and participants

Impact of Notifications on Nutrition, Sleep, and Physical Activity on Intellectual Function and Muscle Mass in Older Adults (INSPIOR) was a single-centre, open-label, randomised controlled trial in a community setting in Kashiwa, Japan. Invitation cards were mailed to all 2210 members aged 65 years or older of A-Shi-Ta, a community health-promotion facility in the Kashiwanoha area, whose membership register was the sampling frame. Many of these members also belong to the Kashiwa Study cohort^15^ in the same area. Respondents telephoned to arrange an explanation and screening visit (Fig. 1). Participants were eligible if they lived independently, were able and willing to wear a Fitbit device for at least 20 h per day, had smartphone access, and had at least one indicator of increased risk: low body-mass index (BMI) for age; pre-frailty or frailty on the FRAIL questionnaire^16^; or sarcopenia-related decline indicated by low grip strength, gait speed of less than 1 m/s, or low skeletal muscle mass index (SMI). Participants were excluded if they were receiving full-time care in a care facility, were already using or had previously used a wearable device to manage their own health, or had uncontrolled hypertension, uncontrolled diabetes, or uncontrolled chronic kidney disease. Full criteria are in Supplementary Table S1.

**Fig. 1:**
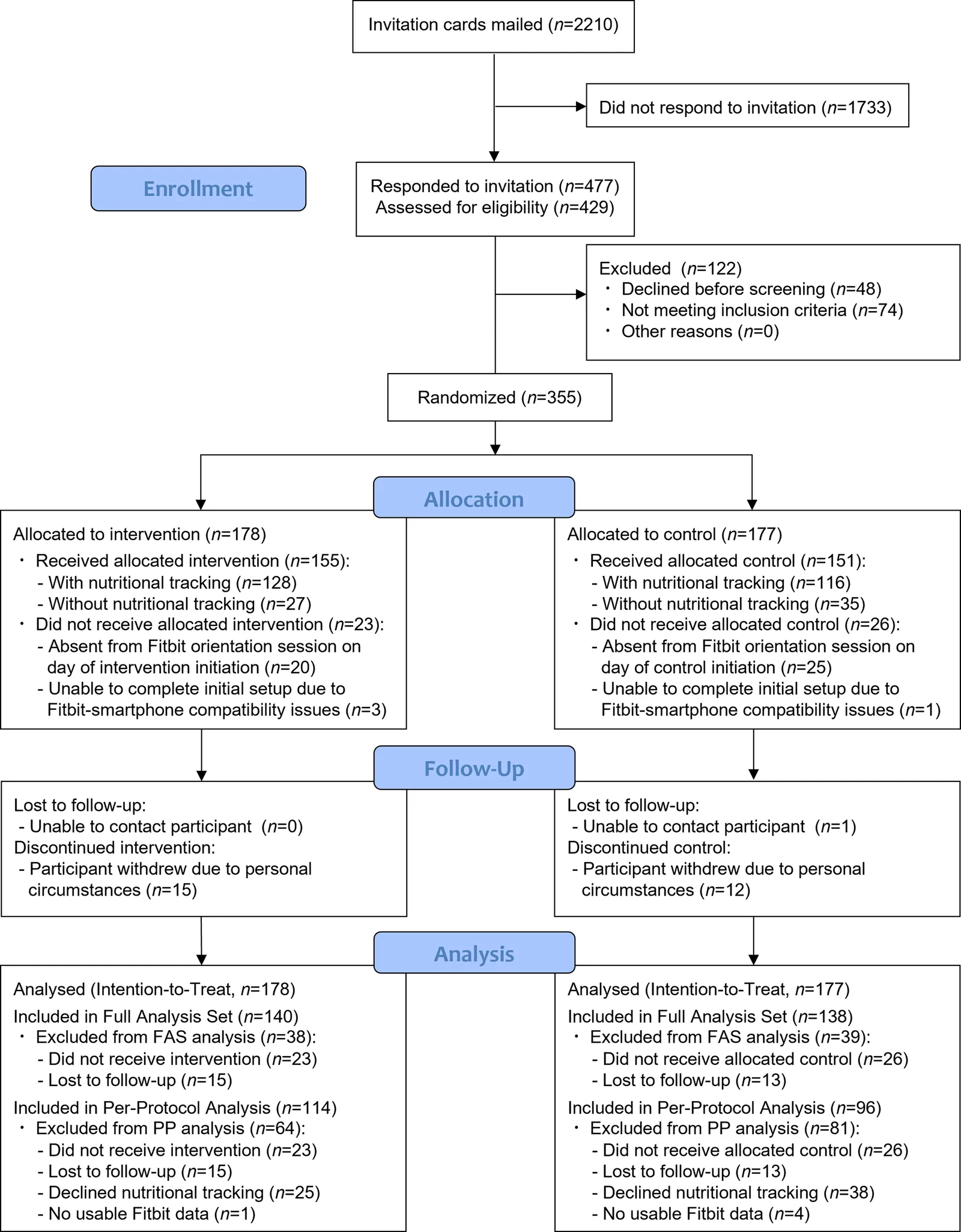
Trial profile.

This trial was prospectively registered with UMIN-CTR (UMIN000052707) on Nov 6, 2023, before the first participant was enrolled on Nov 8, and additionally with ClinicalTrials.gov (NCT06135740; primary outcome measures recorded Nov 30, 2023). This report adheres to CONSORT 2025^17^; the completed CONSORT 2025 checklist is provided as Supplementary Fig. S1 and the CONSORT for Abstracts checklist as Supplementary Fig. S2.

Race and ethnicity were not collected: this single community cohort is ethnically homogeneous. Comorbid conditions were likewise not systematically recorded. Because only uncontrolled hypertension, uncontrolled diabetes, and uncontrolled chronic kidney disease were exclusion criteria, participants whose conditions were controlled remained eligible; their prevalence was not ascertained and cannot be reported by assigned group.

Patients or members of the public were not formally involved in the design, conduct, or analysis of this trial. Individual outcome results were returned to each participant by post, and overall findings will be disseminated to the cohort.

### Ethics

The trial was approved by the Ethics Committee of The University of Tokyo (22–360) and was conducted in accordance with the Declaration of Helsinki. All participants provided written informed consent.

### Randomisation and masking

The allocation sequence was generated in advance by the principal investigator from a random number table, using permuted blocks of four in a 1:1 ratio without stratification. Eligibility assessment, consent, and the screening examination—which also served as the baseline outcome assessment—were completed before any allocation was applied, and the sequence was then applied in a single pass in the fixed order in which participants had been screened. Baseline outcome data were therefore collected before the sequence was applied; the single exception is the baseline Trail Making Test (TMT), which for the reason described below had to be re-administered after allocation.

Allocation concealment was not implemented as a separate procedure: the enrolling investigator also applied the sequence, had access to it, and knew the block size, and no envelope system or central allocation was used. Applying the sequence in a single pass, after every eligibility decision and screening assessment was complete, removed the usual opportunity to exclude a participant after seeing an assignment, but it does not exclude selection bias altogether. We treat the absence of concealment as a limitation (see Discussion); the Supplementary Methods set out what was and was not protected against.

Masking was not achieved after allocation. Participants necessarily knew their group. The staff who performed the 6-month outcome assessments had also set up the devices, contacted participants during the intervention, and managed the study, so they could often recognise participants and infer their group; separating these roles was not feasible in a single-centre trial of this size.

### Procedures

Participants in the intervention group received Fitbit Charge 5 (Google, Mountain View, CA, USA) and were asked to wear it continuously for 6 months, including during sleep. The first 28 days were a baseline measurement window in which no prompt was sent; prompting began one month after wear started and ran for the remaining 5 months. Alerts were generated from each participant's own averaged data on monthly and weekly schedules and delivered as text messages. Targets rose in steps: for daily step count, successive targets of 3000, 6000, and 8000 steps, maintained at 8000 or more; for exercise time, successive weekly targets of 45, 90, and 150 min, the highest of which follows the WHO recommendation for moderate–intensity activity,^18^ maintained at 150 min or more; for sleep, successive targets of 5 h and 6 h per night, with a 6–9 h maintenance band and a caution at 9 h or more; and, in the nutrition component, successive targets of 13% and 15% of energy intake from protein, with a 15–20% maintenance band and a caution at 20% or more. Weekly alerts additionally addressed sleep–wake variability, using a threshold of 30 min, and recognised a 10% or greater increase in step count or exercise time over the preceding week (Supplementary Tables S2 and S3). Prompting was purely threshold-based goal setting: no educational material, counselling, or motivational content was delivered, and no specific behaviour-change theory was used as a framework. The optional nutrition component used the Asken food-management application (Asken, Tokyo, Japan) and was confined to protein intake rather than broader dietary advice. Protein was targeted not because increasing intake improves cognition, but because it was the dietary exposure most directly aligned with the muscle-related vulnerability that defined eligibility, and because a single quantifiable nutrient could be tracked automatically. Participants who opted in could receive protein-balanced foods free of charge (NH Foods, Tokyo, Japan), because increasing protein intake may impose a financial burden. The food was one of the options presented within the protein prompt, so only participants who took up the dietary component could receive it; they collected it at the study facility, and the quantity collected was not recorded. It was not selected from existing products but developed for this trial by a collaborating manufacturer as a meatball-style food combining animal (chicken) and plant (soy) protein, and containing dietary fibre, lipid, and anserine (Supplementary Table S4). “Protein-balanced food” is the manufacturer's product name rather than a defined category, and no evidence exists on its effects on health outcomes in older adults; it was provided as a practical means of increasing protein intake, not as a component expected to act on cognition.

Prompt issuance was tightly coupled to device wear; per-protocol intervention participants wore the Fitbit on a median of 121·8 days (interquartile range [IQR] 87·3–145·5) of 180, counted across the whole wear period including the 28 baseline days before any prompt.

Participants were introduced to the devices at a group orientation and device-setup session, and human contact thereafter was limited to technical support by telephone and, where necessary, in person. The automated prompts were logged: 146 intervention participants were sent a mean of 16·9 weekly activity, 16·9 weekly sleep, 4·9 monthly activity, and 4·9 monthly sleep prompts each, and 118 were sent a mean of 3·5 monthly nutrition prompts (Supplementary Table S20). Delivery was logged but engagement was not: whether a prompt was read or acted on was not recorded, and use of the Fitbit and dietary applications was not measured in either group, so the extent of self-monitoring is unknown. Exposure can therefore be described only as prompts delivered and device worn. The support arrangements, and a registered telephone follow-up procedure that was not implemented, are described in the Supplementary Methods.

The control group received no prompts, advice, education, or other component of the intervention at any time; control participants wore the Fitbit and recorded diet in the Asken application during the baseline and end-of-study assessment windows only. We describe this arm as an assessment-only control rather than as usual care; the trial registration describes it as a sham comparator receiving regular care and health-related information, but no sham procedure was used and neither care nor health-related information was provided to this arm.

A procedural problem confined to the baseline TMT assessment bears on how the TMT estimates should be read. TMT has no official training or certification programme and no authorised Japanese-language script was available to us, and all examiners administered the baseline TMT under the same non-standard procedure, unaware that it differed from the standard one. This was identified before the intervention began, so no 6-month data or treatment-effect estimate existed, and the test was re-administered under the standard procedure, but after allocation, in a group session under time pressure, and with the allocation known to participants and examiners, unlike every other baseline measure. Standard-procedure baseline scores were therefore available for 254 of 355 participants for TMT-A and 253 of 355 for TMT-B, distributed almost identically between the groups (128 of 178 intervention, 127 of 177 control), although those re-assessed were younger and scored higher on the Montreal Cognitive Assessment (MoCA). The TMT estimates are reported in full but interpreted as non-confirmatory for these reasons, and not because the registered plan drew any such distinction; the problem did not affect MoCA, SMI, or the body-composition outcomes. The full account is in the Supplementary Methods; Supplementary Table S14 compares those re-assessed with those not.

MoCA was administered in its validated Japanese version by certified examiners following the published MoCA-J manual and was independently double-scored, whereas no equivalent training, certification, script, or double-scoring was in place for TMT — the context in which the baseline administration problem arose. Inter-rater reliability was not assessed for either instrument and sessions were not recorded (Supplementary Table S11).

Adverse events were collected through spontaneous reports and systematic enquiry at scheduled outcome-assessment visits; definitions are given in the Supplementary Methods.

### Outcomes

Three co-primary outcomes were registered: executive function, measured by TMT parts A and B (completion time in seconds; shorter is better)^19^; global cognition, measured by the Japanese version of the MoCA (range 0–30, higher is better)^20^; and muscle mass, measured as the SMI (kg/m^2^; higher is better). Outcomes were assessed at baseline and 6 months. Cognition and muscle mass were registered as co-primary outcomes rather than either being subordinate; no metabolic muscle–brain axis was posited. No ranking or hierarchy among the three was pre-specified in the registration or protocol, and none was introduced afterwards. All three are reported in full.

Secondary outcomes were the Fitbit-derived daily step count and total sleep time.

Other registered outcomes were frailty index and dietary protein intake; SMI was measured alongside the other body-composition measures (MC-190; Tanita, Tokyo, Japan). No exploratory outcome was pre-specified; unregistered measures are analysed as exploratory throughout. Supplementary Table S10 lists every outcome, its classification in the registered plan, the analysis applied, and each deviation.

Fitbit-derived measures were taken from the Fitbit Web application programming interface; total active time was the fairly active minutes plus twice the very active minutes, and a day contributed only if at least 100 steps were recorded and the activity durations totalled at least 80% of the day. Only the nutrient totals computed by the Asken application were available to the investigators, and intake could not be broken down by food, so consumption of the protein-balanced food itself could not be quantified. Derivations, thresholds, and their limitations are given in Supplementary Table S19.

### Statistics

The target sample size was 350 participants (175 per group); assuming 20% attrition, this left 280 evaluable participants, giving 80% power at a two-sided alpha of 0·05 to detect a standardised effect size of 0·34, a value between Cohen's conventional small and medium benchmarks.^21^ Power was calculated separately for each registered primary outcome from published standard deviations in external reference populations; in natural units, 0·34 corresponds to 0·27 kg/m^2^ for SMI, 9·4 s for TMT-A, 31·3 s for TMT-B, and 1·1 points for MoCA (Supplementary Table S1b). Multiplicity was not incorporated: 80% is the marginal power for each outcome alone, not the joint power of demonstrating an effect on all three.

The intention-to-treat (ITT) population included all randomised participants (178; 177). The full analysis set (FAS) comprised participants with a post-baseline assessment of the outcome analysed, irrespective of completeness of Fitbit or Asken recording; for MoCA this was 140 and 138 (Supplementary Table S6). The per-protocol (PP) population comprised participants who completed the data-collection protocol assigned to their group (114 intervention; 96 control; baseline characteristics by population in Supplementary Table S12): Fitbit use throughout the 6 months in the intervention group and during the two assessment windows in the control group, together with agreement to record diet in the Asken application.

For the clinical outcomes, post-intervention values were analysed by analysis of covariance (ANCOVA) with treatment group as the main effect and baseline outcome value, age, sex, BMI, years of education, and ApoE2 and ApoE4 carrier status as covariates. The set was applied to every clinical outcome and was fixed before any outcome analysis, although not registered, so covariates were not selected per outcome after the data were available. Except for the re-administered baseline TMT, all covariates were measured before randomisation. Missing post-baseline clinical outcomes were imputed by multiple imputation^22^ in R with predictive mean matching, using 50 imputations (m = 50) pooled by Rubin's rules (Supplementary Table S16); the imputation was univariate rather than iterative, and the full specification is in the statistical analysis plan. These analyses assume that data were missing at random conditional on the variables in the imputation model; this assumption cannot be verified.

Fitbit-derived daily logs were analysed in the PP population using generalised linear mixed models (GLMM) comparing the first 28 days of wear, before any prompt was sent, with the final 28 days, during which prompting was still being delivered (specification in the statistical analysis plan).

MoCA was the only one of the three co-primary outcomes to show a between-group difference, so their exploratory analyses — the treatment-by-adherence interaction, the subgroup analyses, the behaviour-association model, and the responder analysis — were all carried out on MoCA. That choice followed the primary results rather than any pre-specified plan, so they are post hoc and unadjusted for multiplicity (Supplementary Table S10).

To evaluate whether the effect on MoCA was larger in adherent participants, we fitted a complete-case ANCOVA in the FAS (n = 278) with a treatment-by-adherence interaction term, adherence being membership of the PP set vs FAS-only (n = 68), covariates matching the primary analysis.

Exploratory PP nutrition outcomes were each participant's mean intake over two 14-day windows: weeks 3–4, immediately before prompting began, and weeks 24–25, near the end of the wear period and after dietary prompting had ended. Means from the follow-up window were analysed by ANCOVA adjusting for the corresponding pre-intervention mean and the primary covariate set.

Exploratory associations between 6-month behaviour change and ΔMoCA were assessed in the PP population in a single ANCOVA entering change in exercise (composite z-score of daily steps and total active time), change in sleep, and change in protein intake simultaneously, adjusted for baseline MoCA, the corresponding pre-intervention values, and the primary covariates.

Exploratory subgroup analyses were restricted to the FAS so that observed ΔMoCA could be used without imputation; comparisons used two-sided Mann–Whitney U tests. No strategy for controlling multiplicity across the three co-primary outcomes was pre-specified, so all reported p values are unadjusted and all subgroup and exploratory analyses are hypothesis-generating; the three co-primary outcomes analysed jointly with Holm adjustment are in Supplementary Table S21. ANCOVA was chosen for the clinical outcomes because it adjusts for the baseline value, generalised linear mixed models for the repeatedly measured device data because they accommodate within-participant correlation, and Mann–Whitney U tests for the subgroup comparisons because normality was not assumed. Sample size, randomisation, masking, the outcomes, and the eligibility criteria are described in the corresponding subsections above.

### Role of the funding source

The study sponsor, the University of Tokyo, had final responsibility for the study. NH Foods funded a University of Tokyo social cooperation programme and supplied the protein-balanced foods; Google provided Fitbit devices and a research donation; Mitsui Fudosan managed the recruitment membership list and provided assessment space. Employees of NH Foods and Google are co-authors and in that capacity contributed to the conception and design of the trial and to the review of the manuscript; beyond that, the funders had no role in the conduct of the trial, data collection, analysis or interpretation, or the writing of the report, and no access to individual-level data. The corresponding author had full access to all study data and final responsibility for the decision to submit.

## Results

Between Nov 8, 2023, and Jul 31, 2024 (primary completion), 355 participants were randomly assigned, 178 to intervention and 177 to control (Fig. 1). Of the 477 people who responded to the invitation, 48 declined before screening and 74 of the 429 assessed for eligibility did not meet the inclusion criteria; no participant was excluded for any other reason. The FAS for MoCA comprised 278 participants (140; 138) and the PP population 210 (114; 96); FAS membership is outcome-specific (Supplementary Table S6). Baseline characteristics were well balanced (Table 1). Baseline MoCA ranged from 13 to 30 (median 25); 183 participants (51·5%) scored below 26, the cut-off for suspected mild cognitive impairment (MCI), and 11 (3·1%) scored the maximum of 30, so the scale was not near its ceiling (Supplementary Table S22).

**Table 1:** Baseline characteristics of randomised participants.

Characteristic	Intervention (n = 178)	Control (n = 177)
Age, years	75·0 (5·3)	75·4 (5·2)
Female sex	116 (65·2%)	117 (66·1%)
Education, years	14·0 (2·3)	14·1 (2·4)
BMI, kg/m^2^	21·5 (2·9)	21·7 (3·2)
ApoE2 carrier	11 (6·2%)	20 (11·3%)
ApoE4 carrier	32 (18·0%)	29 (16·4%)
Suspected MCI	90 (50·6%)	93 (52·5%)
MoCA, score	25·1 (2·7)	25·0 (3·2)
TMT-A, s	85·4 (32·1); n = 128	85·9 (27·5); n = 126
TMT-B, s	123·8 (42·3); n = 126	126·4 (51·0); n = 127
SMI, kg/m^2^	6·6 (1·1)	6·6 (1·1)
Frailty index, score	1·3 (1·0)	1·2 (0·9)

Data are mean (SD) for continuous variables and n (%) for categorical variables. BMI, body-mass index; MCI, mild cognitive impairment; MoCA, Montreal Cognitive Assessment; SMI, skeletal muscle mass index; TMT, Trail Making Test. Suspected MCI was defined as MoCA <26. TMT values are observed completion times in seconds; baseline TMT was re-administered under the standard procedure, after allocation, at the group orientation session described in the Methods and was available for 254 participants for TMT-A and 253 for TMT-B, so the number contributing to each TMT cell is shown beside it. Comorbid conditions such as hypertension and diabetes were not systematically recorded and their prevalence cannot be reported (see Methods).

In the ITT analysis of MoCA, one of three co-primary outcomes, the adjusted between-group difference favoured the intervention (0·58 points, 95% CI 0·03–1·13; p = 0·039, unadjusted; Fig. 2). Analysed jointly with the other two co-primary outcomes and adjusted by the Holm procedure, the p value for MoCA was 0·12 and the difference was not statistically significant (Supplementary Table S21).

**Fig. 2:**
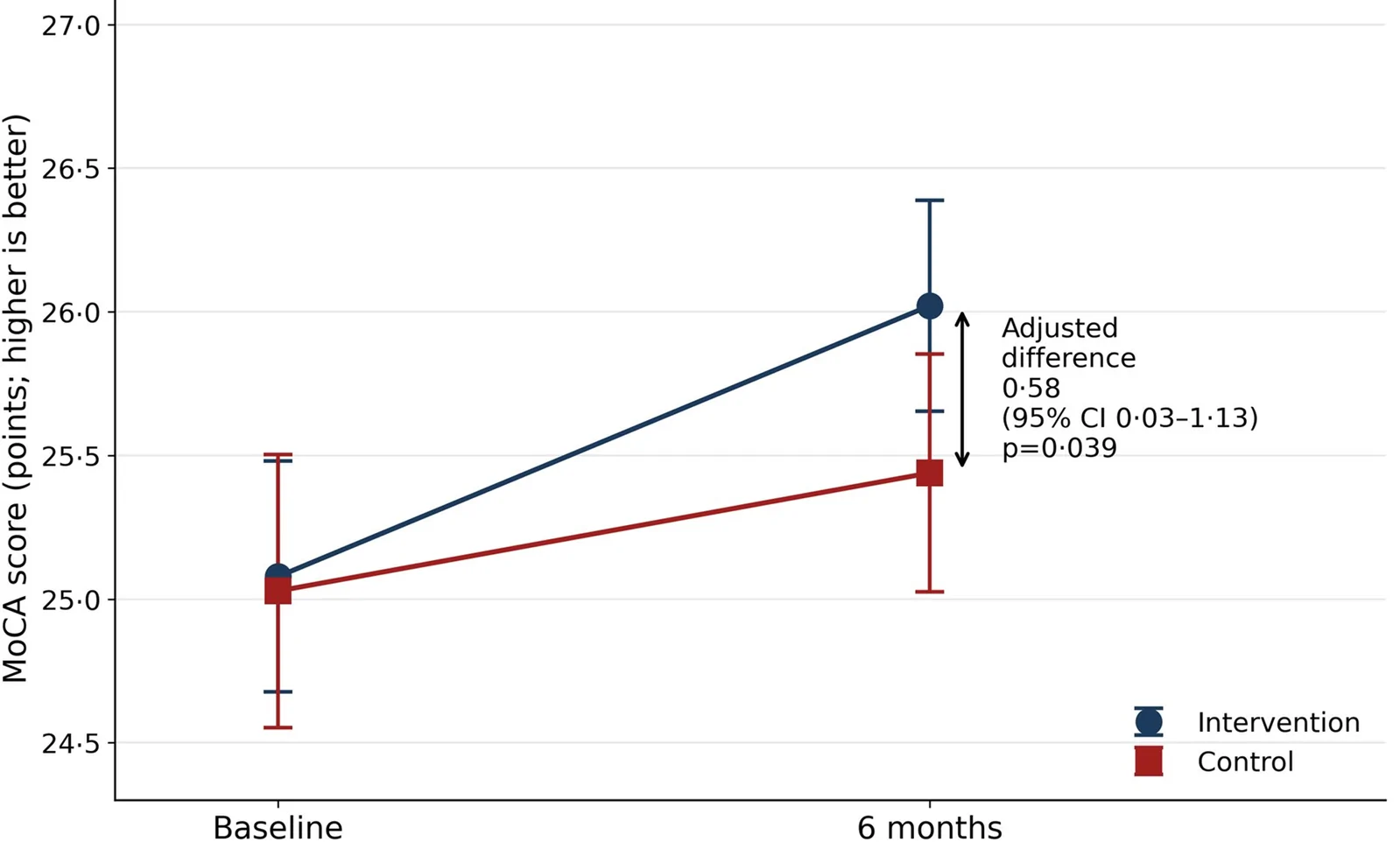
MoCA in the intention-to-treat population. Error bars show 95% CIs. Baseline values are observed group means; 6-month values and the annotated between-group difference are from the MI ANCOVA primary analysis. MoCA, Montreal Cognitive Assessment; MI, multiple imputation; ANCOVA, analysis of covariance.

TMT scores showed no between-group difference: 0·16 s for TMT-A (95% CI −5·07 to 5·40; p = 0·95) and 5·59 s for TMT-B (−3·88 to 15·05; p = 0·25) in the ITT analysis, and likewise non-significant in the FAS and PP populations (Supplementary Table S6).

No clear difference was observed for SMI (adjusted difference −0·01 kg/m^2^, 95% CI −0·09 to 0·07; p = 0·88) or for the body-composition measures. The frailty index showed a modest difference favouring the intervention group (−0·19, −0·37 to −0·01; p = 0·043; Supplementary Table S6).

Sensitivity analyses across the three analysis populations were directionally consistent, with somewhat larger adjusted differences in the complete-case FAS and PP populations (0·61 and 0·92 points; Fig. 3; Supplementary Table S5). An ANCOVA in the FAS with a treatment-by-adherence interaction term gave a significant interaction (1·60, 95% CI 0·39–2·82; p = 0·010). Baseline characteristics did not differ materially between PP and FAS-only participants and the interaction persisted after additional adjustment (Supplementary Table S13); because adherence is a post-randomisation variable, this analysis is exploratory and non-causal. In a post-hoc responder analysis in the FAS, more intervention than control participants improved on MoCA by at least 1 point (84/140 [60·0%] vs 57/138 [41·3%]), 2 points (58/140 [41·4%] vs 37/138 [26·8%]), and 3 points (42/140 [30·0%] vs 18/138 [13·0%]; Supplementary Table S15); these thresholds were neither pre-specified nor established as clinically important.

**Fig. 3:**
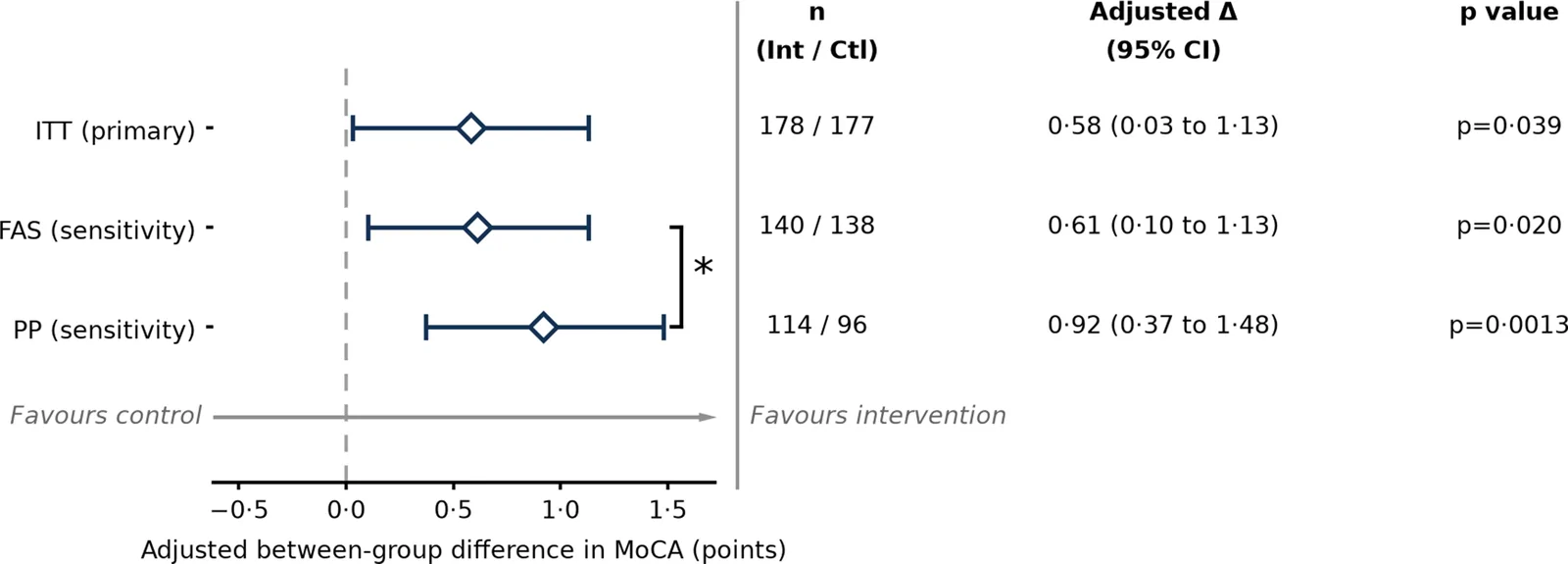
Sensitivity analyses of post-intervention MoCA across analysis sets. Forest plot of the adjusted between-group difference in MoCA in the ITT, FAS, and PP populations. The vertical bracket between the FAS and PP rows, marked with ∗, denotes a statistically significant (p < 0·05) treatment × adherence interaction in the FAS-based ANCOVA (PP vs FAS-only; coefficient 1·60, 95% CI 0·39–2·82; p = 0·010). MoCA, Montreal Cognitive Assessment; ITT, intention-to-treat; FAS, full analysis set; PP, per-protocol; ANCOVA, analysis of covariance.

In GLMM analyses of Fitbit-derived daily logs in the PP population, which carry the two registered secondary outcomes, group-by-period interactions were significant for steps, active time, and sleep time (Fig. 4; Supplementary Table S7). In natural units the between-group differences in change were 696 steps per day, 6·6 min of active time per day, and 11·6 min of sleep per night; for steps and sleep both groups declined and the difference reflects a smaller decline rather than an increase, whereas active time rose in the intervention group and was unchanged in the control group (Supplementary Table S23).

**Fig. 4:**
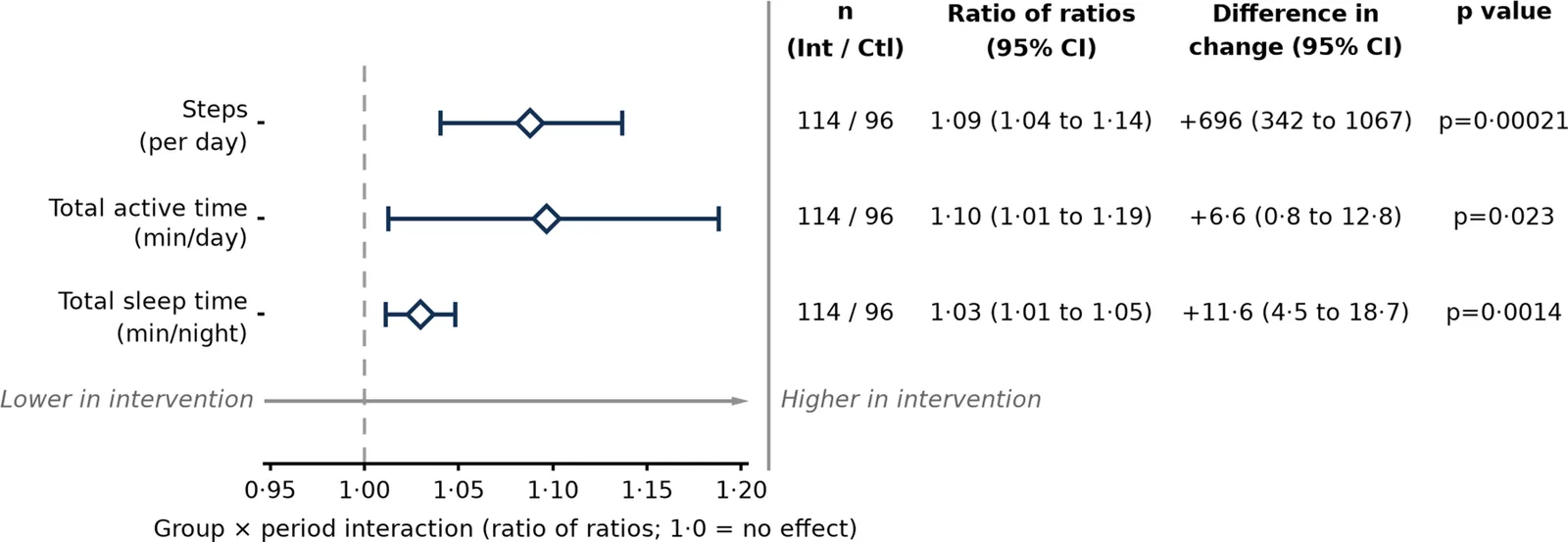
Fitbit-derived daily behaviours in the per-protocol population. Each row reports the group-by-period interaction (ratio of ratios) from a generalised linear mixed model on continuously recorded daily lifelog data. A value of 1·0 indicates no between-group difference in the relative change from baseline; values above 1·0 favour the intervention. The difference in change expresses the same contrast in natural units — steps per day, minutes per day, and minutes per night, as given in each row label — back-transformed from the estimated marginal means of the same models. Its confidence interval was obtained by applying the confidence limits of the group-by-period interaction to the estimated marginal means, so it carries the uncertainty of the interaction term and not that of the estimated means themselves. These models include no covariates, so this contrast is not covariate-adjusted and is not comparable with the adjusted differences in Figs. 3 and 5. Supplementary Table S23 reports the estimated means in each window, by group.

In exploratory PP nutrition analyses, the intervention increased protein intake (adjusted difference 7·97 g/day, 95% CI 4·90–11·03; p < 0·0001) and, to a smaller degree, lipid, dietary fibre, and total energy intake; carbohydrate intake did not clearly differ (Fig. 5; Supplementary Table S8). Of the 178 assigned to the intervention, 128 (71·9%) took up the optional dietary component and 121 of them (94·5%) also opted to receive the protein-balanced food, as did 109 of the 114 per-protocol intervention participants. How much each participant collected or ate was not recorded.

**Fig. 5:**
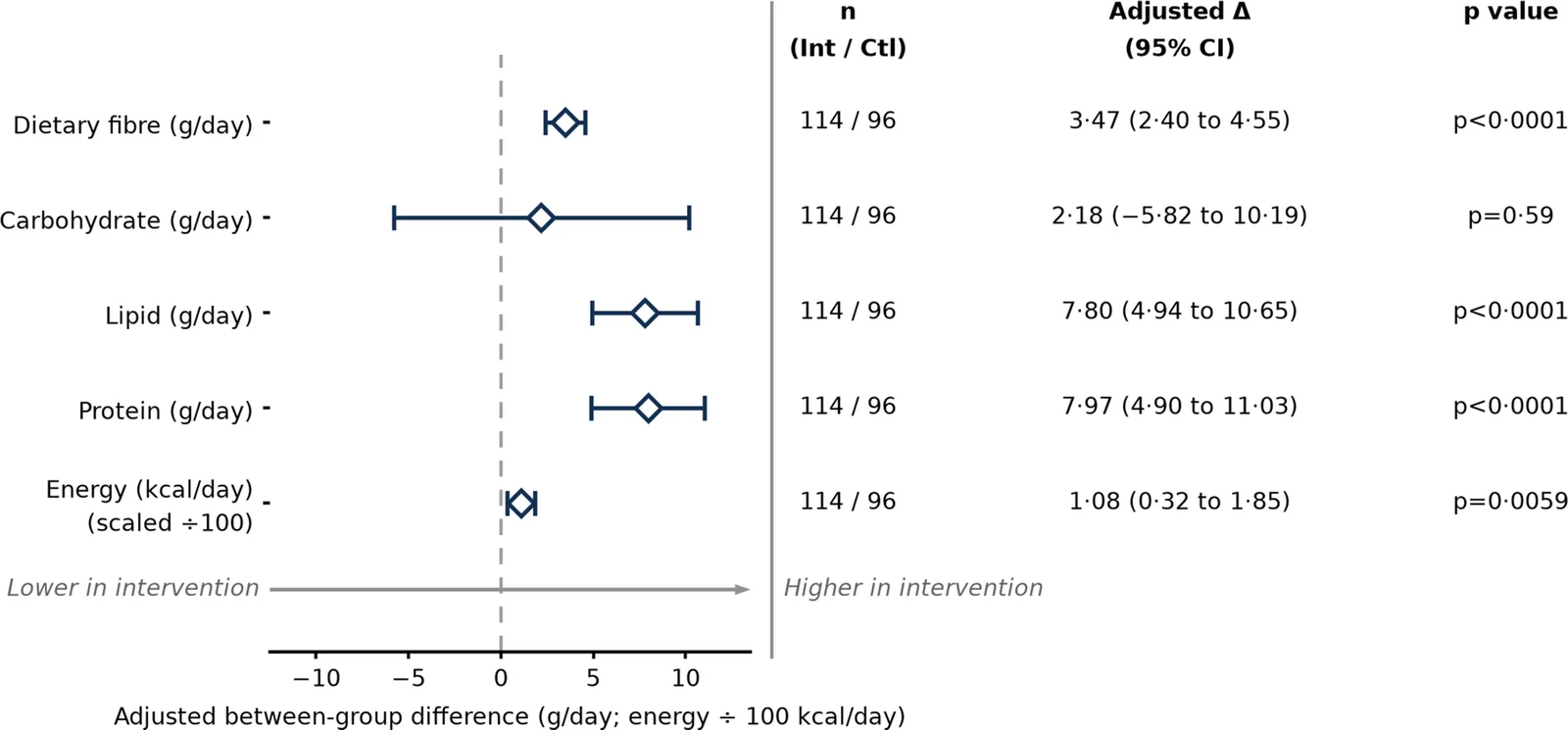
Macronutrient and dietary-fibre outcomes in the per-protocol population. Forest plot of the adjusted between-group difference at weeks 24–25, after dietary prompting had ended, estimated by ANCOVA. Energy is rescaled (÷100) so it shares an axis with the gram-per-day outcomes. Models adjust for the corresponding pre-intervention value and the prespecified covariates. ANCOVA, analysis of covariance.

In the exploratory change-score model described in the Methods, greater increases in exercise were associated with larger improvements in ΔMoCA (standardised β = 0·24, p = 0·0086). Neither change in sleep (β = 0·16, p = 0·36) nor change in protein intake (β = −0·03, p = 0·87) was retained (Fig. 6; Supplementary Table S9). The activity, sleep, and nutrition components were delivered together and the nutrition component was optional, so the trial cannot attribute the difference in MoCA to any one of them.

**Fig. 6:**
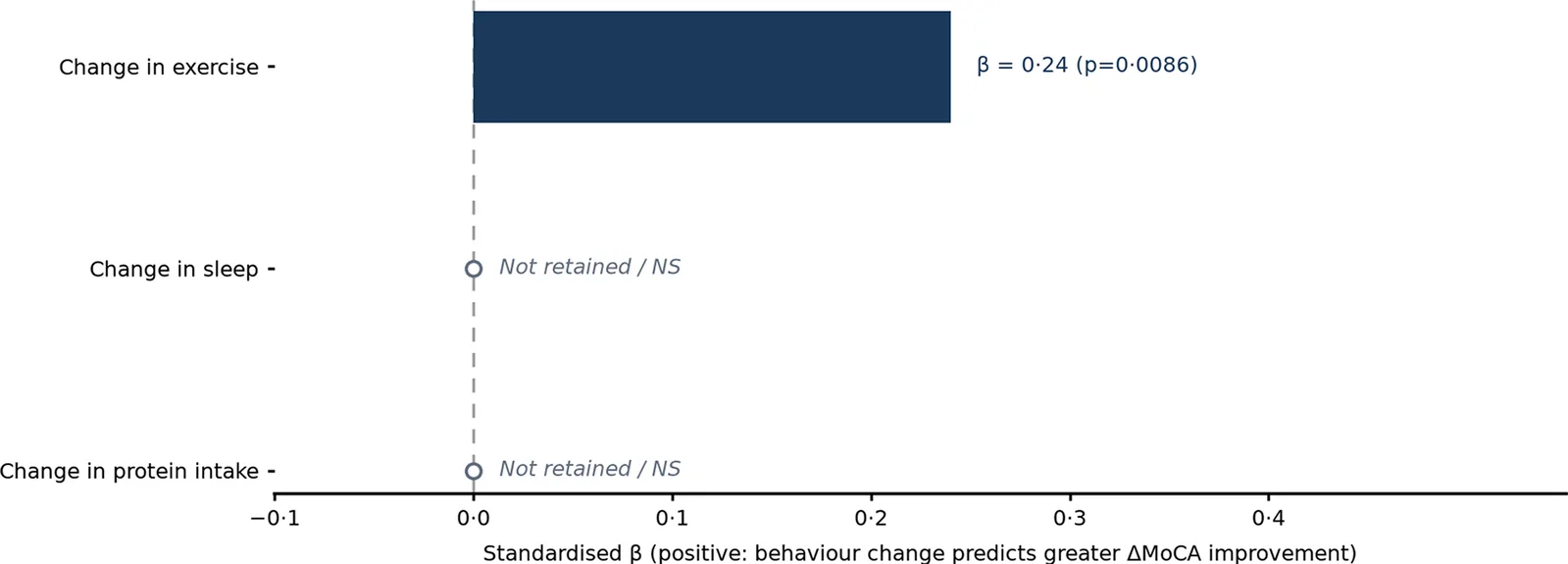
Exploratory associations between 6-month behaviour change and *Δ*MoCA in the per-protocol population. A single ANCOVA model regressed post-intervention MoCA on three behaviour-change predictors entered simultaneously — change in exercise (composite z-score of daily steps and total active time), change in sleep (total sleep time), and change in protein intake — together with baseline MoCA, the corresponding pre-intervention behaviour values and the prespecified covariates. Bars show the standardised regression coefficient (*β*) for predictors retained at p < 0·05; open circles mark predictors that were not retained. Positive *β* values mean that greater behaviour change predicted greater *Δ*MoCA improvement. Full model coefficients are reported in Supplementary Table S9. ANCOVA, analysis of covariance; MoCA, Montreal Cognitive Assessment.

Formal tests of effect modification gave no evidence that the effect differed by baseline cognitive status (treatment-by-suspected-MCI interaction p = 0·92), by ApoE4 carriership (p = 0·43), or by their combination (p = 0·33). The largest apparent separation, among participants with both suspected MCI and ApoE4 carriership (n = 7 vs 10; p = 0·038), rests on very small numbers, is uncorrected for multiplicity, is not supported by the corresponding interaction test, and is not interpretable for clinical inference (Fig. 7; Supplementary Table S18).

**Fig. 7:**
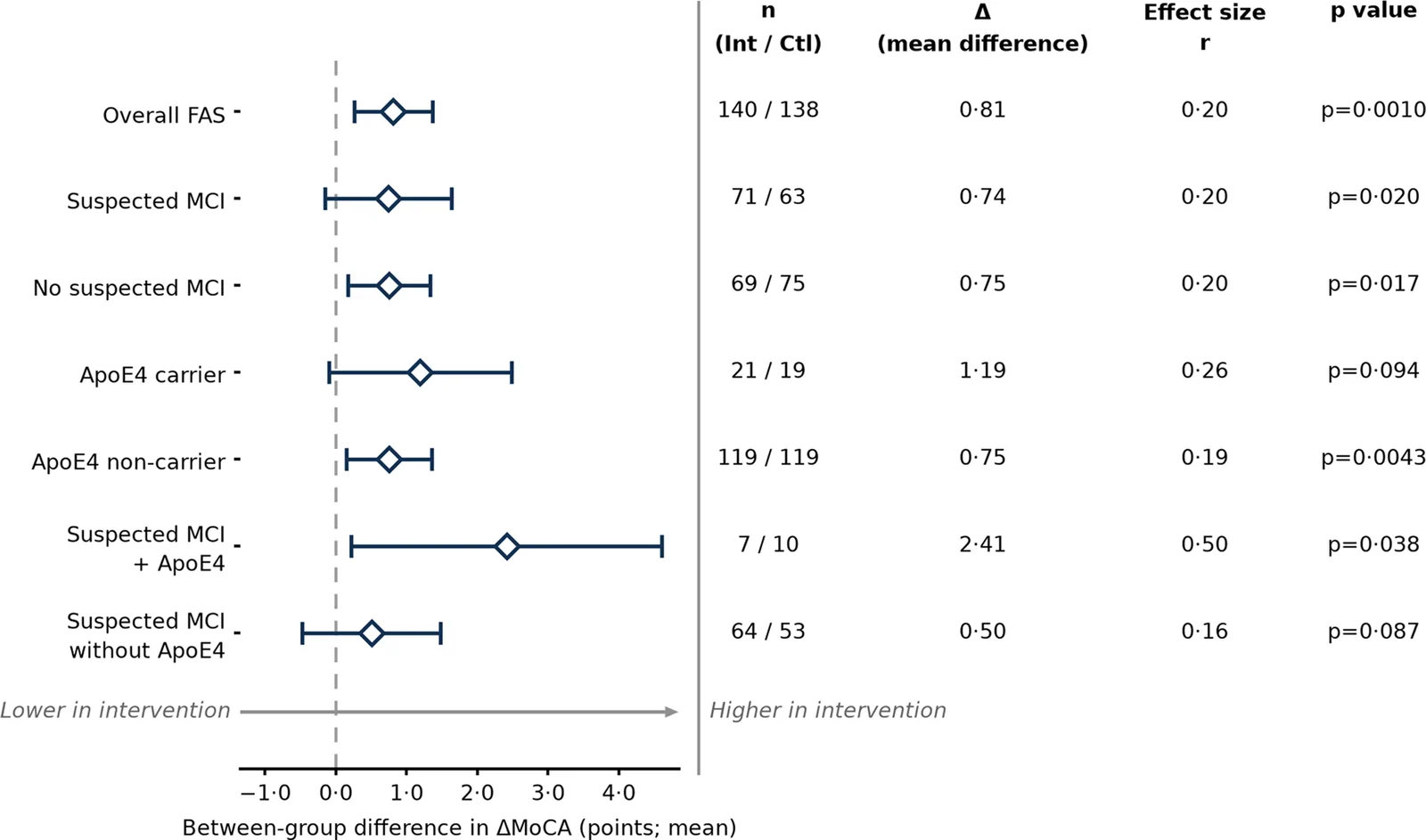
Exploratory subgroup analyses of ΔMoCA in the full-analysis-set population. Subgroup comparisons were performed using two-sided Mann–Whitney U tests. Each row shows the between-group mean difference in *Δ*MoCA, an approximate 95% CI based on the observed SD and per-arm *n*, the rank-based effect size *r*, and the unadjusted p value. All subgroup results are exploratory and uncorrected for multiplicity. Because of very small subgroup sizes (as few as seven vs 10 participants) and the absence of multiplicity adjustment, these subgroup results are not interpretable for clinical inference.

No serious adverse events related to the device, the nutrition application, or the protein-balanced food were reported in either group. Non-serious events possibly related to the intervention were mild skin irritation at the site of Fitbit contact and stress associated with daily dietary recording; a small number of control participants also reported mild skin irritation. No participant withdrew because of an intervention-related adverse event. Usability scores at 6 months were similar in the two groups, though no formal comparison was made (Supplementary Table S17).

## Discussion

In this trial, an automated, wearable-based multidomain lifestyle intervention was associated with a small difference in global cognition (MoCA) compared with an assessment-only control.

Previous trials have relied on substantial human support. INSPIOR shows that an analogue whose behavioural content is generated and delivered algorithmically, with human contact limited to setup and technical support, can also show a measurable cognitive signal. Wearable trials have repeatedly improved physical activity^7^ but rarely tested cognition.

The intervention group took more daily steps, accumulated more total active time, and slept slightly longer, in the directions reported for step counts,^10^ exercise,^11^ and sleep duration^12^ with dementia risk, and with randomised work in which automated coaching increased physical activity at scale.^23^ Only the intervention group wore the device continuously, so part of these differences may reflect reactivity to measurement rather than the prompts. In an exploratory model entering all three behaviour-change domains at once, only change in exercise was associated with change in MoCA. These changes are best read as evidence of engagement rather than of the pathway through which any cognitive change occurred: the model is observational, rests on behavioural measures recorded under different conditions in the two groups, and cannot support a mediation interpretation. The dietary component was optional, intake of the protein-balanced food itself was not measured, no comparison with other protein sources was made, and change in protein intake was not associated with change in MoCA. Baseline intakes were close to age- and sex-matched national means (Supplementary Tables S8 and S8b), so this was intake above the average rather than correction of a shortfall.^24^

No minimal clinically important difference for the MoCA has been universally accepted, and estimates vary by method.^25^ Our estimate lies well below the minimal detectable change reported for the MoCA in a comparable population (MDC95 4 points),^26^ a threshold that describes the reliability of one person's change score, whereas our estimate is a group-mean contrast. The distribution-based minimal clinically important difference is 1·0 point^25^; compatible with the upper bound of our confidence interval (1·13) but not with the point estimate. We therefore read the finding not as a clinically meaningful improvement for an individual but as a small shift in the population distribution of cognitive scores, of uncertain clinical significance. Such a shift need not be inconsequential: in an observational cohort, scoring 29 rather than 30 on a 30-point instrument was associated with a higher rate of recorded dementia diagnosis (adjusted hazard ratio 1·43, 95% CI 1·11–1·84).^27^ That baseline comparison cannot establish what an intervention-induced 0·58-point change means. These comparisons are in the Supplementary Discussion.

The subgroup differences in Fig. 7 should not be read as identifying groups that benefit more, notwithstanding evidence that ApoE4 status can modify the response to lifestyle factors^28^; they rest on small numbers, are uncorrected for multiplicity, and are hypothesis-generating; the prognosis of mild cognitive impairment is itself heterogeneous.^29^ The treatment-by-adherence interaction is similarly exploratory: adherence is post-randomisation, so the larger per-protocol estimate is not evidence of a stronger biological effect. The effect was not replicated on the Trail Making Test. We cannot distinguish whether the baseline administration problem degraded that analysis or the intervention had no effect on this domain; either way, the absence of corroboration on a second instrument weakens the case that the difference reflects a broad cognitive benefit.

Allocation concealment was not implemented, so selection bias is not excluded, and 0·58 MoCA points is small enough that even modest bias would matter; the Supplementary Methods detail what was and was not protected against. Outcome assessment at 6 months was also not masked, so measurement, expectancy, and scoring effects cannot be excluded. The quality-control procedures for MoCA reduce but do not remove this concern, and the effect estimate should be read as correspondingly uncertain.

INSPIOR was conducted at a single centre in suburban Japan, and 6 months cannot speak to longer-term durability. The behavioural and dietary outcomes were measured while prompting was still being delivered, so they describe behaviour under active prompting rather than behaviour that persisted after it stopped. The MoCA result is one of three co-primary analyses rather than a single confirmatory finding; considered jointly, it is no longer statistically significant. The trial was powered on 280 evaluable participants; 278 contributed to the full analysis set for MoCA and 210 met the per-protocol criteria, so per-protocol comparisons are less well powered than planned; the intention-to-treat analysis remains the primary inference. Comorbidity data were not collected, so we cannot report the prevalence of conditions such as hypertension and diabetes or examine them as effect modifiers. Assumed and observed standard deviations differed, most markedly for TMT-B (Supplementary Table S1b), so precision is better judged from the reported confidence intervals than from planned power. Reactivity to being measured is a recognised source of the behavioural differences described above.^30^

Participants owned a smartphone and were willing to wear a wrist-worn device for 6 months; people already using a wearable to manage their own health were excluded, so the findings describe introducing such a device rather than continuing with one; race and ethnicity were not recorded because the population is ethnically homogeneous. Socioeconomic position was not measured beyond years of education, and disability was not assessed; eligibility relied on validated measures of physical function and adults in full-time institutional care were excluded, so the findings speak little to older adults with substantial functional limitation or in different social circumstances. Sex was included as a covariate in every outcome model, but the trial was not powered to estimate sex-specific effects and outcomes are not reported separately for women and men, who made up two-thirds of the participants. How far they extend to rural or lower-resource settings, or to older adults with lower digital literacy, is unknown. Scalability is further qualified by the technical support needed, which we did not record, and the automated design may make the programme less effective where digital access or familiarity is limited.

Overall, INSPIOR supports the feasibility of scalable, automated, wearable-based multidomain programmes for healthy ageing. Larger and longer multicentre trials are needed to establish whether the MoCA difference is durable and clinically consequential and which components contribute. These data inform short-term cognitive-score change in a physically risk-enriched older cohort; they do not directly inform dementia prevention.

## Contributors

Conceptualisation: KS, YS, JL, SW, and TH. Methodology: KS, YS, JL, and TH. Formal analysis: KS, IS, H Kasahara, SA, RT, MW, MF, and TH. Investigation: KS, IS, H Kasahara, SA, RT, MW, MF, SS, TG, RK, KM, TK, and TH. Resources: NI, K Kaneta, S Kobayashi, NK, TM, TT, TI, K Kudo, YS, H Kayama, JL, and TH. Data curation: KS, IS, H Kasahara, SA, RT, MW, MF, SS, TG, RK, and TH. Writing — original draft: KS, RT, and TH. Writing — review and editing: all authors. Supervision: TH. TH had full access to all the data in the study and had final responsibility for the decision to submit for publication. KS, RT, and TH directly accessed and verified the underlying data reported in the manuscript. All authors read and approved the final version of the manuscript.

## Data sharing statement

Deidentified individual participant data will be considered for sharing with academic researchers on submission of a methodologically sound proposal, beginning 12 months after publication and ending 5 years after publication, subject to institutional approvals, ethical constraints, and a signed data-access agreement. The study protocol and the statistical analysis plan are provided with this article and will be available with it; the informed consent form is available from the corresponding author on the same terms. The analysis code will be considered for sharing on the same terms and through the same process, and provided on request. The statistical models, the covariate set, the analysis populations, and the handling of missing data are described in the Methods, the statistical analysis plan, and the appendix. Proposals should be directed to the corresponding author by email.

## Declaration of interests

TH leads a University of Tokyo social cooperation programme funded by NH Foods Ltd. NK, TM, TT, TI, K Kudo, and YS are employees of NH Foods Ltd. JL, H Kayama, and MJT are employees of Google or Google LLC. NI, K Kaneta, S Kobayashi, and TN are affiliated with Mitsui Fudosan, which supported recruitment logistics and provided some assessment space. All other authors declare no competing interests.

## Appendix A Supplementary data

Appendix

Study protocol

Statistical analysis plan
