# Supplementary material for: Effect of a wearable-based individualised multidomain lifestyle intervention on cognitive and physical function in older adults at risk of decline (INSPIOR): a single-centre, open-label, randomised controlled trial
Source: eClinicalMedicine. 2026 Sep 23;100:104212. doi: 10.1016/j.eclinm.2026.104212 (PMC13634265; doi:10.1016/j.eclinm.2026.104212)
Supplement: Supplementary file 1 [file mmc1.pdf]

Appendix

Effect of a wearable-based individualised multidomain lifestyle intervention on cognitive and physical function in older adults at risk of decline (INSPIOR): a single-centre, open-label, randomised controlled trial

Note on references and numbering. *Numeric citations in this Appendix refer to the reference list of the main manuscript and are listed there in full. Citations prefixed with SR (SR1, SR2, ...) refer to the Supplementary references at the end of this Appendix. Supplementary tables are cited as table S1, table S2, and so on; the two series are numbered independently.*

Contents

Supplementary Methods

Method 1. Administration of the baseline Trail Making Test

Method 2. Randomisation, allocation concealment, and masking

Method 3. Eligibility criteria as applied

Method 4. Device support, prompt delivery, the control condition, and adverse events

Supplementary Discussion

Supplementary Tables S1–S23

Supplementary Figure 1. CONSORT 2025 checklist

Supplementary Figure 2. CONSORT for Abstracts checklist

Supplementary references SR1–SR12

Supplementary Methods

This section gives in full the methodological detail summarised in the Methods of the main manuscript.

Method 1. Administration of the baseline Trail Making Test

Unlike the MoCA, the Trail Making Test has no official training or certification programme, and no authorised Japanese-language administration script was available to us. The procedure the examiners followed did not have them point out sequencing errors; instead they recorded the number of errors. Under the standard procedure the examiner corrects errors in real time while timing continues, so that the completion time includes the time taken to correct them. All examiners administered the baseline

TMT under the same non-standard procedure, unaware that it differed from the standard one; this was not a matter of variation between examiners.

The discrepancy was identified at an investigators' meeting by a co-investigator who was not an examiner and had previously administered the test. This occurred after every screening assessment had been completed but before the orientation and device-setup session at which the intervention began, so no participant had yet received any part of the intervention and no 6-month outcome data or treatment-effect estimates existed. TMT was therefore re-administered under the standard procedure at that session. The session was not one-to-one: 40–50 participants attended at a time, several staff circulated among them, and time was limited, so participants whose device setup took longer could not be re-assessed; some who had attended screening did not attend at all. Because the content of the session differed between the two groups, both participants and examiners were aware of the allocation when the TMT was re-administered. Because the session was the first day of the intervention, no later occasion for a pre-intervention measurement existed.

The originally recorded non-standard scores are retained but are not comparable with standard-procedure scores and were not analysed. The same version of the test and the same test forms were used at every administration, so the repeated baseline scores and the 6-month scores carry practice effects from the administrations that preceded them; participants who were re-assessed took the same forms three times and those who were not took them twice, and the schedule of administrations was the same in both groups. The 6-month TMT was administered one-to-one under the standard procedure, concurrently with the other 6-month outcomes. Participants without a standard-procedure baseline score were retained in the intention-to-treat analyses: the model adjusted for baseline TMT where available and for the remaining covariates where it was not.

The baseline scores used in the TMT analyses were thus obtained under the correct procedure but after allocation, in a group setting under time pressure and with allocation known to participants and examiners — unlike every other baseline measure, which preceded randomisation — and they are missing for a selected subset. Participants without a standard-procedure baseline score were older and scored lower on MoCA than those with one, so the TMT analyses describe a younger and cognitively better preserved subset of the randomised population. The proportion re-assessed was nevertheless almost identical in the two groups (128 of 178 intervention, 127 of 177 control), and within that subset baseline characteristics, including TMT itself, were similar between groups, so the loss is unlikely to have acted differentially on the between-group contrast. There was no evidence that re-assessment was related to the date of the screening visit. Supplementary table S14 gives the comparison in full, and the identity of the examiner was not recorded for individual assessments, so re-assessment cannot be compared across examiners.

Method 2. Randomisation, allocation concealment, and masking

The allocation sequence was generated in advance by the principal investigator from a random number table, using permuted blocks of four in a 1:1 ratio. Allocation concealment was not implemented as a separate procedure. The investigator who enrolled participants also applied the allocation sequence, had access to it, and was aware of the block size; no envelope system, central allocation, or other safeguard against prediction of the next assignment was used.

Applying the sequence in a single pass, after every eligibility decision and screening assessment was complete, removed the usual opportunity to exclude a participant after seeing an assignment, and it means that every baseline measure except the re-administered TMT was recorded before any allocation was applied. But it does not exclude selection bias altogether: the enrolling investigator knew the sequence and the block size, nothing prevented that knowledge from influencing the order in which participants were screened, and the assignment of the last participants in each block was predictable in principle.

Masking was not achieved after allocation. Participants necessarily knew their group, and the staff who performed the 6-month outcome assessments had also set up the devices, contacted participants during the intervention, and managed the study, so they could often recognise participants and infer their group. Separating these roles was not feasible in a single-centre trial of this size.

Method 3. Eligibility criteria as applied

The criteria reproduced in Supplementary table S1 were applied as follows. The indicators of increased risk were a BMI below 20 kg/m² for participants aged 65–69 years or below 21·5 kg/m² for those aged 70 years or older; pre-frailty or frailty on the FRAIL questionnaire, a five-item self-report tool covering Fatigue, Resistance, Ambulation, Illnesses, and Loss of weight; or sarcopenia-related decline indicated by low grip strength, gait speed below 1 m/s, or low skeletal muscle mass index. At least one of these was required.

In addition to the exclusions given in the Methods, participants were excluded if they were unable to receive or act on the study interventions, had a cardiac pacemaker or cardiovascular stent, had none of the risk indicators above, or had any other condition that the principal investigator considered unsuitable for participation.

Method 4. Device support, prompt delivery, the control condition, and adverse events

Participants were introduced to the devices at the group orientation and device-setup session described in the Methods. During the intervention, participants who had difficulty with the device or the application, or whose device malfunctioned, were supported first by telephone; when a problem could not be resolved by telephone, a member of the university research team visited the facility and helped in person. The number of telephone and in-person support contacts was not systematically recorded.

The trial registration also described telephone follow-up of participants who did not change their behaviour after an alert or who were not wearing the device. This was not implemented, because staffing allowed only for responding to problems that participants themselves reported. No participant was contacted because of their behavioural data.

The assessment-only control group received no prompts, advice, education, or other component of the intervention at any time. They wore the Fitbit and recorded diet in the Asken application during the baseline and end-of-study assessment windows only. Use of ordinary community and municipal health services was neither influenced nor recorded in either group.

Adverse events were defined as any untoward medical occurrence reported by participants during the trial, including device-related skin irritation, sleep disturbance attributed to prompt notifications, and psychological distress associated with dietary recording. Serious adverse events were defined according to ICH-GCP criteria and reported to the Ethics Committee of The University of Tokyo.

Supplementary Discussion

Interpreting a between-group difference of 0·58 MoCA points

The reading given in the Discussion rests on the following comparisons. Thresholds derived for the MoCA describe the reliability of one person's change score, whereas our estimate is a group-mean contrast, and the two are not interchangeable. Retest and practice effects are of a similar order to our estimate, but they would arise in both arms and largely cancel in a between-group contrast, as would regression to the mean under baseline-adjusted ANCOVA; because assessors were not masked, we cannot assume that they were identical in the two groups.

A post-hoc responder analysis describes the same result in distributional terms, and is reported in Supplementary table S15. It uses thresholds of 1, 2, and 3 MoCA points, none of which was pre-specified and none of which corresponds to an established threshold for a clinically important change; all three are reported so that no threshold appears to have been selected after the fact.

The observational comparison cited in the Discussion, in which older adults scoring 29 rather than 30 on a 30-point instrument had a higher long-term rate of recorded dementia diagnosis, uses a different instrument from the MoCA and concerns the baseline score rather than an intervention-induced change. It is given to show that a difference of this size need not be inconsequential at the population level, not as an estimate of what 0·58 MoCA points would mean.

Supplementary Tables

Supplementary Table S1. Detailed eligibility criteria for study participants

Inclusion criteria

Category	Criteria
**Age**	Aged 65 years or older and less than 93 years.
**Living status**	Living in Japan; community-dwelling and living independently (with or without carer support), and not requiring nursing care provided by long-term care insurance.
**Technology**	Ability and willingness to wear a Fitbit device for the study duration (≥20 hours/day, including during sleep); smartphone access for Fitbit app installation and data synchronisation.
**Consent**	Capacity to provide written informed consent.
**Comorbidity (low BMI)**	BMI <20 kg/m² for ages 65–69 years, or BMI <21·5 kg/m² for ages ≥70 years.
**Physical decline (frailty)**	FRAIL questionnaire[16] score of 1–2 (pre-frailty) or ≥3 (frailty).
**Physical decline (sarcopenia)**	Low muscle strength (grip strength <18 kg female, <28 kg male), low physical performance (gait speed <1·0 m/s), or low muscle mass (SMI <5·7 kg/m² female, <7·0 kg/m² male).

Exclusion criteria

Category	Criteria
**Residence**	Residence in a full-time care facility.
**Medical conditions**	Uncontrolled hypertension, uncontrolled diabetes, or uncontrolled chronic kidney disease; presence of cardiac pacemakers or cardiovascular stents.
**Prior wearable use**	Existing wearable users, or prior experience using a wearable to manage their own health.
**Other**	Inability to receive or act on study interventions; any other condition deemed inappropriate for participation by the Principal Investigator; absence of any of the specified inclusion criteria related to comorbidities or physical decline.

BMI, body mass index; SMI, skeletal muscle mass index. At least one of the comorbidity (low BMI) or physical-decline indicators (frailty or sarcopenia) was required in addition to the age, living-status, technology, and consent criteria. Frailty was assessed using the FRAIL questionnaire. Eligibility criteria were designed to enrich the study population for individuals at elevated risk of cognitive and physical decline.

Supplementary Table S1b. Sample-size calculation: assumptions, their sources, and the target treatment effect in the natural units of each primary outcome

Primary outcome	Assumed SD	Source of the assumed SD	Target difference at d=0·34	Observed baseline SD in this trial
**Skeletal muscle mass**	0·8	Fukuoka et al. [SR1]	0·27 kg/m²	1·10

**index, kg/m²**				
**Trail Making Test A, s**	27·5	Hashimoto et al. [SR2]	9·4 s	29·9
**Trail Making Test B, s**	92·0	Hashimoto et al. [SR2]	31·3 s	46·8
**Montreal Cognitive Assessment, points**	3·11	Nasreddine et al. [SR3]	1·1 points	2·98

The trial was designed to randomise 350 participants (175 per group). Assuming 20% attrition, 280 participants (140 per group) would remain evaluable, giving 80% power at a two-sided alpha of 0·05 to detect a standardised effect size of 0·34 — a value lying between Cohen's conventional small (0·20) and medium (0·50) benchmarks[21]. The 20% attrition figure was a conservative design assumption and was not derived from a specific external estimate. The assumed standard deviations were taken from published external reference populations whose composition differs from that of this trial; the observed baseline standard deviations are shown for comparison only and were not used to revise the pre-specified calculation. Baseline covariates, which increase precision, were ignored in the calculation, making it conservative. Power was computed separately for each registered primary outcome; multiplicity across the three co-primary outcomes, and the joint probability of demonstrating an effect on all three, were not incorporated into the sample size.

Supplementary Table S2. Intervention alert logic based on Fitbit data

Monthly alerts

Alert type	Criterion (current state)	Message content	Target state
**Step count**	Average daily steps <3000	Encourage to increase steps	≥3000
**Step count**	3000 ≤ average daily steps <6000	Encourage to increase steps	≥6000
**Step count**	6000 ≤ average daily steps <8000	Encourage to increase steps	≥8000
**Step count**	Average daily steps ≥8000	Congratulate; encourage to maintain	≥8000
**Exercise time**	Weekly total exercise time <45 min	Encourage to increase exercise time	≥45 min
**Exercise time**	45 ≤ weekly total exercise time <90 min	Encourage to increase exercise time	≥90 min
**Exercise time**	90 ≤ weekly total exercise time <150 min	Encourage to increase exercise time	≥150 min
**Exercise time**	Weekly total exercise time ≥150 min	Congratulate; encourage to maintain	≥150 min
**Sleep duration**	Average daily sleep <5 hours	Encourage to increase sleep duration	≥5 hours
**Sleep duration**	5 ≤ average sleep <6 hours	Encourage to increase sleep duration	≥6 hours

**Sleep duration**	6 ≤ average sleep <9 hours	Congratulate; encourage to maintain	6 ≤ sleep <9 hours
**Sleep duration**	Average daily sleep ≥9 hours	Oversleep caution	6 ≤ sleep <9 hours

Weekly alerts

Alert type	Criterion (current state)	Message content	Target state
**Step count**	Average daily steps ≥8000	Congratulate; encourage to maintain	≥8000
**Step count**	Average daily steps <8000 and step count increased by ≥10% versus the prior week	Congratulate; encourage to improve	≥10% week-over-week increase
**Step count**	Average daily steps <8000 and change <10%	Encourage to improve	≥10% week-over-week increase
**Exercise time**	Weekly total exercise time ≥150 min	Congratulate; encourage to maintain	≥150 min
**Exercise time**	Weekly total exercise time <150 min and increased by ≥10% versus prior week	Congratulate; encourage to improve	≥10% week-over-week increase
**Exercise time**	Weekly total exercise time <150 min and change <10%	Encourage to improve	≥10% week-over-week increase
**Sleep duration**	Average daily sleep <5 hours	Encourage to increase sleep duration	≥5 hours
**Sleep duration**	5 ≤ average sleep <6 hours	Encourage to increase sleep duration	≥6 hours
**Sleep duration**	6 ≤ average sleep <9 hours	Congratulate; encourage to maintain	6 ≤ sleep <9 hours
**Sleep duration**	Average daily sleep ≥9 hours	Oversleep caution	6 ≤ sleep <9 hours
**Sleep/wake variability**	Average bedtime or wake-time variability <30 min	Congratulate; encourage to maintain	<30 min
**Sleep/wake variability**	Average bedtime or wake-time variability ≥30 min	Encourage to improve	<30 min

Fitbit-derived data were processed monthly and weekly to determine appropriate alerts, which were delivered as SMS messages to participants. The step-count and sleep criteria are daily averages over the preceding period. The exercise-time criterion is a weekly total, formed by multiplying the average daily active time over the preceding period by seven; the thresholds of 45, 90, and 150 min therefore apply to the week and not to the day. They were set with reference to the World Health Organization recommendation that adults, including older adults, undertake at least 150 min of moderate-intensity aerobic physical activity per week.[18]

Supplementary Table S3. Nutritional intervention alert logic based on nutritional tracking data

Alert type	Criterion (current state)	Message content	Target state
**Monthly protein intake**	Average ratio of protein-energy intake to daily energy consumption <13%	Encourage to increase protein intake	≥13%
**Monthly protein intake**	13% ≤ ratio <15%	Encourage to increase protein intake	≥15%
**Monthly protein intake**	15% ≤ ratio <20%	Congratulate; encourage to maintain	15–20%
**Monthly protein intake**	Ratio ≥20%	Over-protein caution	15–20%

Nutritional data were collected through the Asken food-management application and processed monthly to determine appropriate alerts, delivered as SMS messages to participants. Protein-intake thresholds were derived from the methodology of the Kawasaki Aging and Wellbeing Project.[SR4]

Supplementary Table S4. Nutritional composition and ingredients of the protein-balanced food

Nutrient	Amount per 100 g
**Energy (kcal)**	229·0
**Protein (g)**	17·1
**Fat (g)**	15·0
**Carbohydrates (g)**	8·3
**Net carbohydrate (g)**	4·5
**Dietary fibre (g)**	3·8
**Sodium (mg)**	565·0
**Salt equivalent (g)**	1·4
**Water content (g)**	57·4
**Ash content (g)**	2·2
**Anserine (mg)**	179·6
**Carnosine (mg)**	55·9

Ingredients

Chicken, granular soybean protein, vegetable oil, onion, breadcrumbs, bonito seasoning, kelp extract, ginger, reduced starch syrup, salt, soy sauce, refined sugar, frying oil, seasoning (amino acids, etc.), methylcellulose, acidulant, xylose, flavouring, phosphate.

Composition is presented per 100 g, which corresponds to the recommended daily intake. The food was developed for this trial by the manufacturer; "protein-balanced food" is a product name and not a defined category. Anserine and carnosine are naturally occurring constituents of chicken[SR5] and are reported here for completeness of the compositional analysis; no claim is made about their contribution to any outcome of this trial.

Supplementary Table S5. Sensitivity analyses of post-intervention MoCA across analysis sets

Analysis set	Total n	Int / Ctl	Adjusted Δ (points)	95% CI	p value
**ITT (primary)**	355	178 / 177	0·58	0·03 to 1·13	0·039
**FAS (sensitivity)**	278	140 / 138	0·61	0·10 to 1·13	0·020
**PP (sensitivity)**	210	114 / 96	0·92	0·37 to 1·48	0·0013

Sensitivity analyses of post-intervention MoCA across the ITT, FAS, and PP populations.

Supplementary Table S6. Clinical outcomes analysed with ANCOVA

Analysis set	Outcome	Unit	n (Int / Ctl)	Adjusted Δ	95% CI	p value
**ITT**	TMT-A	s	178 / 177	0·16	−5·07 to 5·40	0·95
**FAS**	TMT-A	s	128 / 126	−0·72	−6·17 to 4·72	0·79
**PP**	TMT-A	s	106 / 89	−1·04	−7·36 to 5·29	0·75
**ITT**	TMT-B	s	178 / 177	5·59	−3·88 to 15·05	0·25
**FAS**	TMT-B	s	126 / 126	3·27	−6·73 to 13·27	0·52
**PP**	TMT-B	s	104 / 89	1·97	−9·65 to 13·60	0·74
**ITT**	SMI	kg/m^2	178 / 177	−0·01	−0·09 to 0·07	0·88
**FAS**	SMI	kg/m^2	140 / 138	−0·00	−0·09 to 0·08	0·92
**PP**	SMI	kg/m^2	114 / 96	0·00	−0·11 to 0·11	1·0
**ITT**	Muscle mass	kg	178 / 177	0·14	−0·11 to 0·38	0·27
**FAS**	Muscle mass	kg	140 / 138	0·12	−0·15 to 0·38	0·39
**PP**	Muscle mass	kg	114 / 96	0·10	−0·19 to 0·39	0·49
**ITT**	Body weight	kg	178 / 177	−0·18	−0·56 to 0·20	0·35
**FAS**	Body weight	kg	140 / 138	−0·14	−0·55 to 0·26	0·49
**PP**	Body weight	kg	114 / 96	0·26	−0·22 to 0·74	0·28
**ITT**	Body fat percentage	%	178 / 177	0·08	−0·53 to 0·69	0·80
**FAS**	Body fat percentage	%	140 / 138	0·05	−0·59 to 0·68	0·89

**PP**	Body fat percentage	%	114 / 96	0·46	−0·18 to 1·09	0·16
**ITT**	BMI	kg/m^2	178 / 177	−0·07	−0·23 to 0·08	0·36
**FAS**	BMI	kg/m^2	140 / 138	−0·06	−0·21 to 0·10	0·45
**PP**	BMI	kg/m^2	114 / 96	0·08	−0·10 to 0·26	0·37
**ITT**	Frailty index	score	178 / 177	−0·19	−0·37 to −0·01	0·043
**FAS**	Frailty index	score	140 / 138	−0·19	−0·37 to −0·01	0·044
**PP**	Frailty index	score	114 / 96	−0·27	−0·47 to −0·07	0·010

Clinical outcomes by analysis set.

Supplementary Table S7. Fitbit-derived daily logs analysed by GLMM

Outcome	Ratio of ratios	95% CI	p value
**Steps**	1·09	1·04 to 1·14	0·00021
**Total active time**	1·10	1·01 to 1·19	0·023
**Total sleep time**	1·03	1·01 to 1·05	0·0014

Estimates are the group × period interaction (ratio of ratios) from generalised linear mixed models with random intercepts at the participant level in the per-protocol population (n=210; 114 intervention, 96 control).

Supplementary Table S8. Exploratory PP nutrition outcomes

Outcome	Baseline intervention	Baseline control	n analysed (Int / Ctl)	Adjusted Δ	95% CI	p value
**Energy, kcal/day**	1724·4 (353·1)	1664·0 (295·1)	114 / 96	108·1	31·5 to 184·6	0·0059
**Protein, g/day**	74·0 (18·5)	71·1 (13·5)	114 / 96	7·97	4·90 to 11·03	<0·0001
**Lipid, g/day**	59·6 (14·9)	57·4 (13·7)	114 / 96	7·80	4·94 to 10·65	<0·0001
**Carbohydrate, g/day**	231·7 (47·1)	224·4 (42·1)	114 / 96	2·18	−5·82 to 10·19	0·59
**Dietary fibre, g/day**	22·7 (6·3)	21·3 (5·2)	114 / 96	3·47	2·40 to 4·55	<0·0001

All intake values are derived from the Asken food-management application and are means of the recorded assessment window. These outcomes were exploratory and were not registered. Reference intakes in the general Japanese population are given in table S8b.

Supplementary Table S8b. Reference intakes in the general Japanese population, for comparison with table S8

Group	Energy, kcal/day	Protein, g/day	Lipid, g/day	Carbohydrate, g/day	Dietary fibre, g/day
**Men aged 65–74**	2166	79·6	67·3	274·8	20·1

**(n=461)**					
**Women aged 65–74 (n=508)**	1719	67·5	56·6	227·1	18·8
**Men aged ≥75 (n=415)**	1998	74·7	58·8	269·1	20·7
**Women aged ≥75 (n=499)**	1687	65·5	51·0	235·6	19·2

Mean daily intakes in the 2023 Japanese National Health and Nutrition Survey.[24] Weighting these strata to the age and sex distribution of the participants with an observed baseline nutrition record (101 intervention, 75 control) gives reference values of 1848 kcal/day energy, 70·7 g/day protein, 57·7 g/day lipid, 246·0 g/day carbohydrate, and 19·5 g/day dietary fibre for the intervention group, and 1840, 70·4, 57·3, 245·6, and 19·5 for the control group. The national estimates come from a single weighed household dietary record, whereas the values in table S8 are application-derived means in a selected per-protocol sample at one site; they are therefore descriptive external benchmarks and are not directly comparable, and no statistical comparison was made.

Supplementary Table S9. Exploratory associations between 6-month behaviour change and ΔMoCA (per-protocol population)

Predictor	Standardised β	SE	p value
**MoCA_pre**	−1·019	0·155	<0·0001
**Change in exercise**	0·235	0·088	0·0086
**Exercise_pre**	−0·092	0·081	0·26
**Change in sleep**	0·159	0·173	0·36
**Sleep_pre**	0·037	0·157	0·81
**Change in protein intake**	−0·029	0·178	0·87
**Protein_pre**	0·259	0·184	0·16
**Age**	−0·367	0·154	0·018
**Male**	−0·495	0·355	0·17
**Years of education**	−0·316	0·156	0·044
**ApoE2**	0·063	0·534	0·91
**ApoE4**	0·212	0·400	0·60
**BMI**	0·366	0·152	0·017

Model fit: Adjusted R^2 = 0·234; overall regression p-value = 2×10^{-8}.

Single ANCOVA model fitted in the per-protocol population; specification follows the primary MoCA analysis. "Change in exercise" is a composite z-score of the change in daily steps and the change in total active time. "Change in sleep" is the change in total sleep time. "Change in protein intake" is the 6-month change in daily protein intake. Exercise_pre, Sleep_pre, and Protein_pre are the corresponding pre-intervention values. All analyses are exploratory and uncorrected for multiplicity.

Supplementary Table S10. All trial outcomes, their classification in the registered plan, the analyses applied, and deviations from the registered plan

Outcome	Classification in the registered plan	Analysis applied	Reported in
**Executive function — Trail Making Test (TMT-A, TMT-B)**	Co-primary (no ranking pre-specified)	ITT ANCOVA with multiple imputation; FAS and PP complete-case	Results; table S6
**Global cognition — Montreal Cognitive Assessment (MoCA)**	Co-primary (no ranking pre-specified)	ITT ANCOVA with multiple imputation; FAS and PP complete-case	Results; Fig. 2, 3; table S5
**Muscle mass — skeletal muscle mass index (SMI)**	Co-primary (no ranking pre-specified)	ITT ANCOVA with multiple imputation; FAS and PP complete-case	Results; table S6
**Daily step count (Fitbit-derived)**	Secondary	GLMM, group × period interaction, PP	Results; Fig. 4; table S7
**Total sleep time (Fitbit-derived)**	Secondary	GLMM, group × period interaction, PP	Results; Fig. 4; table S7
**Frailty index**	Other registered outcome	ITT ANCOVA with multiple imputation	Results; table S6
**Dietary protein intake**	Other registered outcome	ANCOVA, PP	Results; Fig. 5; table S8
**Total active time (Fitbit-derived)**	Not registered — exploratory	GLMM, group × period interaction, PP	Fig. 4; table S7
**Energy, lipid, carbohydrate and dietary fibre intake**	Not registered — exploratory	ANCOVA, PP	Fig. 5; table S8
**Muscle mass, body weight, body-fat percentage, BMI**	Not registered — exploratory	ITT ANCOVA with multiple imputation	Table S6
**System Usability Scale (SUS)**	Not registered — exploratory	Descriptive	Supplementary table S17
**Treatment-by-adherence interaction on MoCA**	Not registered — exploratory	Complete-case ANCOVA in the FAS	Results; Fig. 3

**Association of 6-month behaviour change with ΔMoCA**	Not registered — exploratory	Single ANCOVA with three predictors, PP	Results; Fig. 6; table S9
**Subgroups of ΔMoCA (suspected MCI, ApoE2, ApoE4, MCI × ApoE4)**	Not registered — exploratory	Two-sided Mann–Whitney U tests, FAS	Results; Fig. 7
**Serious adverse events**	Safety	Descriptive	Results

Classification follows the trial registration (UMIN-CTR UMIN000052707; ClinicalTrials.gov NCT06135740) and the protocol. Three outcomes were registered as primary — TMT, MoCA, and SMI — with no ranking among them and no strategy for controlling multiplicity across them.

Deviations from the registered analysis plan. (1) No hierarchy among the three co-primary outcomes was pre-specified, and none was introduced: all three were analysed by the same intention-to-treat ANCOVA and are reported in full. MoCA was the only one of the three co-primary outcomes to show a between-group difference, so their exploratory analyses — the treatment-by-adherence interaction, the subgroup analyses, the behaviour-association model, and the responder analysis — were all carried out on MoCA. That choice was made after the primary analyses had been seen and is therefore data-dependent; the analyses concerned are reported as exploratory and without multiplicity adjustment. (2) TMT is reported in full but interpreted as non-confirmatory because the baseline assessment was initially administered by all examiners under a non-standard procedure and had to be repeated after allocation, in a group setting and with group assignment known to participants and examiners, leaving it available for a selected subset of participants; this was identified after the baseline assessments had been completed but before the intervention began, and not after the treatment effects had been inspected. (3) The covariate set for the primary ANCOVA and the multiple-imputation framework were fixed by the study team before outcome analysis but were not registered. (4) The specific composition of the ITT, FAS, and PP populations was determined during data analysis and manuscript preparation. (5) No strategy for controlling multiplicity was pre-specified; all p values reported in the manuscript are unadjusted.

Multiplicity. No strategy for controlling multiplicity across the three co-primary outcomes was pre-specified, so all p values reported in the manuscript are unadjusted. Supplementary table S21 reports the three registered co-primary outcomes analysed jointly with Holm adjustment across the intention-to-treat, full-analysis-set, and per-protocol populations; in the intention-to-treat analysis the adjusted p value for MoCA is 0·117 and the difference is not statistically significant.

Supplementary Table S11. Quality-control procedures applied to the cognitive outcome assessments

Quality-control element	MoCA	TMT
**Instrument version**	Validated Japanese version (MoCA-J)	Standard TMT-A and TMT-B; the same version and the same test forms at every administration

**Examiner training and certification**	Official online training and certification programme (MoCA Cognition), completed by every examiner	None; no official training or certification programme exists
**Standardised administration script**	MoCA-J instruction manual, version 2.2 [SR6]	None; no authorised Japanese-language script was available
**Independent double-scoring**	All protocols	None
**Formal inter-rater reliability**	Not assessed	Not assessed
**Audio or video recording of sessions**	Not recorded	Not recorded

Quality-control procedures were applied to MoCA but not to TMT. This asymmetry is the context in which the baseline TMT administration problem described in the Methods arose: with no official programme and no authorised Japanese-language script, the procedure initially adopted proved to be non-standard, and the absence of these safeguards was not identified as a deficiency until that problem came to light. The document from which that procedure was taken has not been identified and cannot be cited. The baseline TMT was consequently re-administered after allocation, unlike every other baseline measure. None of these procedures was applied retrospectively, and no element listed as absent was reconstructed after the fact.

Supplementary Table S12. Baseline characteristics by analysis population

Characteristic	ITT intervention (n=178)	ITT control (n=177)	FAS intervention (n=140)	FAS control (n=138)	PP intervention (n=114)	PP control (n=96)
**Age, years**	75·0 (5·3)	75·4 (5·2)	74·2 (5·2)	74·7 (5·0)	74·2 (5·0)	74·5 (4·9)
**Education, years**	14·0 (2·3)	14·1 (2·4)	14·1 (2·3)	14·2 (2·5)	14·1 (2·4)	14·3 (2·4)
**BMI, kg/m2**	21·5 (2·9)	21·7 (3·2)	21·6 (2·9)	21·9 (3·1)	21·4 (3·0)	21·8 (3·1)
**MoCA, score**	25·1 (2·7)	25·0 (3·2)	25·2 (2·6)	25·6 (2·9)	25·4 (2·5)	25·6 (2·7)
**TMT-A, s**	85·4 (32·1); n=128	85·9 (27·5); n=126	85·4 (32·1); n=128	85·9 (27·5); n=126	85·5 (33·5); n=106	83·7 (23·0); n=89
**TMT-B, s**	123·8 (42·3); n=126	126·4 (51·0); n=127	123·8 (42·3); n=126	126·4 (51·0); n=127	123·9 (42·7); n=104	121·6 (47·5); n=90

**SMI, kg/m2**	6·6 (1·1)	6·6 (1·1)	6·7 (1·1)	6·7 (1·1)	6·7 (1·1)	6·7 (1·2)
**Frailty index, score**	1·3 (1·0)	1·2 (0·9)	1·3 (0·9)	1·2 (0·8)	1·2 (0·8)	1·1 (0·8)
**Female sex**	116 (65·2%)	117 (66·1%)	90 (64·3%)	84 (60·9%)	73 (64·0%)	60 (62·5%)
**ApoE2 carrier**	11 (6·2%)	20 (11·3%)	9 (6·4%)	17 (12·3%)	7 (6·1%)	11 (11·5%)
**ApoE4 carrier**	32 (18·0%)	29 (16·4%)	21 (15·0%)	19 (13·8%)	17 (14·9%)	16 (16·7%)
**Suspected MCI**	90 (50·6%)	93 (52·5%)	71 (50·7%)	63 (45·7%)	53 (46·5%)	45 (46·9%)

Data are mean (SD) for continuous variables and n (%) for categorical variables. ITT, intention to treat (all randomised participants); FAS, full analysis set (participants with a post-baseline assessment of the outcome being analysed; the numbers shown here are those for MoCA); PP, per protocol (participants who completed the data-collection protocol assigned to their group). The three populations are nested. This table is provided so that selection into the analysis sets can be assessed.

Supplementary Table S13. Comparison of per-protocol and full-analysis-set-only participants, and robustness of the treatment-by-adherence interaction

Characteristic	Per protocol (n=210)	FAS only (n=68)	p value
**Age, years**	74·3 (4·9)	74·8 (5·6)	0·57
**Education, years**	14·2 (2·4)	13·9 (2·4)	0·40
**BMI, kg/m2**	21·6 (3·0)	22·2 (2·9)	0·13
**MoCA, score**	25·5 (2·6)	25·1 (3·2)	0·32
**SMI, kg/m2**	6·7 (1·1)	6·7 (1·1)	0·66
**Frailty index, score**	1·2 (0·8)	1·4 (1·0)	0·064
**Female sex**	133 (63·3%)	41 (60·3%)	0·65
**ApoE2 carrier**	18 (8·6%)	8 (11·8%)	0·43
**ApoE4 carrier**	33 (15·7%)	7 (10·3%)	0·27
**Suspected MCI**	98 (46·7%)	36 (52·9%)	0·37
**Treatment-by-adherence interaction**	—	—	
**Primary covariate set**	1·60 points	0·39 to 2·82	0·010
**+ baseline frailty index**	1·58 points	0·36 to 2·80	0·011
**+ baseline frailty index and suspected MCI**	1·60 points	0·38 to 2·82	0·010

Data are mean (SD) for continuous variables and n (%) for categorical variables. p values are from Welch t tests for continuous variables and chi-square tests for categorical variables, and are provided for descriptive purposes only. FAS only denotes participants in the full analysis set who did not meet the per-protocol criteria.

The lower rows report the treatment-by-adherence interaction on post-intervention MoCA under progressively wider covariate adjustment, where adherence is membership in the per-protocol set. Adherence is a post-randomisation variable, so this analysis is exploratory and cannot be interpreted causally; participants who complete a programme may differ from those who do not in ways that are not captured by the measured covariates.

Supplementary Table S14. Baseline characteristics of participants who were and were not re-assessed for baseline TMT

Characteristic	Re-assessed (n=255)	Not re-assessed (n=100)
**Intervention / control**	128 / 127	50 / 50
**Age, years**	74·5 (5·0)	77·0 (5·5)
**Female sex**	160 (62·7%)	73 (73·0%)
**Education, years**	14·1 (2·4)	13·9 (2·3)
**BMI, kg/m2**	21·8 (3·0)	21·1 (3·2)
**ApoE4 carrier**	37 (14·5%)	24 (24·0%)
**Suspected MCI**	124 (48·6%)	59 (59·0%)
**MoCA, score**	25·4 (2·8)	24·2 (3·4)
**SMI, kg/m2**	6·7 (1·1)	6·4 (0·9); n=99
**Frailty index, score**	1·2 (0·8)	1·4 (1·1)
**Contributed to the full analysis set**	255 (100%)	23 (23·0%)

Data are mean (SD) for continuous variables and n (%) for categorical variables. Re-assessed participants are those with a valid baseline score on TMT-A or TMT-B following re-administration at the group orientation and device-setup session; 254 had TMT-A and 253 had TMT-B. Participants were not re-assessed either because they did not attend that session or because the time remaining after device setup did not permit it. Because the content of the session differed between groups, allocation was known to both participants and examiners at the time of re-administration; the resulting baseline scores were nevertheless similar between groups (Table 1). The proportion re-assessed was almost identical in the two groups (128 of 178 in the intervention group and 127 of 177 in the control group), and within the re-assessed subset the baseline characteristics of the two groups, including TMT-A and TMT-B, were similar (Table 1); the subset therefore differs from the rest of the cohort but not between the arms, which makes distortion of the between-group comparison less likely. Selection into re-assessment was nevertheless strongly related to age, MoCA score, ApoE4 status, and retention, so residual bias cannot be excluded. Participants who could be re-assessed were nevertheless younger, scored higher on MoCA, were less often ApoE4 carriers, and almost all went on to contribute outcome data, whereas most of those who were not re-assessed left the trial before the 6-month assessment. The TMT analyses therefore rest on a selected subset of the randomised population, which is a further reason for treating them as non-confirmatory. The same

version of the test and the same test forms were used at every administration: the initial baseline, the repeated baseline, and the 6-month assessment. Participants who were re-assessed therefore took the same forms three times and those who were not took them twice, so both the repeated baseline scores and the 6-month scores carry practice effects. The distribution of the number of administrations was almost identical in the two groups, so the repetition does not act differentially on the between-group contrast. The identity of the examiner was not recorded, so re-assessment cannot be compared across examiners; this is consistent with the absence of a formal inter-rater reliability assessment for either cognitive instrument (Supplementary table S11). Whether a participant could be re-assessed was in any case determined by attendance at the group orientation session and by the time remaining after device setup, not by who was to administer the test. The date of each screening visit was recorded. In a univariate logistic regression with re-assessment as the outcome and the date of the screening visit as the predictor, the odds ratio was 1·08 per 30 days (95% CI 0·96 to 1·23; p=0·198). The interval is compatible with a relation between the period of recruitment and re-assessment, so the test gives no evidence of one rather than evidence of none. The order of participants within a day was not retained, so the sequence cannot be examined at finer than daily resolution.

Supplementary Table S15. Post-hoc responder analysis of MoCA at 6 months

Analysis	Threshold	Intervention	Control	Risk difference (95% CI)	p value	NNT
**FAS, unadjusted**	≥1 point	84/140 (60·0%)	57/138 (41·3%)	18·7 pp (7·1 to 30·2)	0·0018	5·3
**FAS, unadjusted**	≥2 points	58/140 (41·4%)	37/138 (26·8%)	14·6 pp (3·6 to 25·6)	0·010	6·8
**FAS, unadjusted**	≥3 points	42/140 (30·0%)	18/138 (13·0%)	17·0 pp (7·5 to 26·4)	0·00059	5·9
**FAS, adjusted**	≥1 point	—	—	15·8 pp (4·4 to 27·3)	0·0068	6·3
**FAS, adjusted**	≥2 points	—	—	11·4 pp (1·0 to 21·9)	0·032	8·8
**FAS, adjusted**	≥3 points	—	—	14·2 pp (5·0 to 23·4)	0·0024	7·0
**ITT, multiple imputation**	≥1 point	—	—	14·4 pp (3·6 to 25·2)	0·0092	6·9
**ITT, multiple imputation**	≥2 points	—	—	10·6 pp (0·4 to 20·7)	0·041	9·5
**ITT, multiple imputation**	≥3 points	—	—	12·1 pp (3·1 to 21·1)	0·0084	8·3

Responders are participants whose MoCA score increased by at least the stated number of points between baseline and 6 months. Adjusted estimates are risk differences from linear probability models containing treatment group, baseline MoCA, and the covariates of the primary analysis, with heteroscedasticity-consistent

(HC3) standard errors. The intention-to-treat rows use the same multiple-imputation specification as the primary analysis, dichotomising the change score within each imputed dataset and pooling risk differences by Rubin's rules; counts are not shown because they depend on the imputed values. pp, percentage points; NNT, number needed to treat.

These thresholds were not pre-specified and were not derived in this population. No threshold for a clinically important change on MoCA is established: reported minimal detectable change values for an individual cluster between 3 and 5 points, whereas in a stroke cohort assessed over 6 months a distribution-based estimate of a minimal clinically important difference was 1·0 point and an anchor-based estimate was 2·0 points, the latter derived from only 13 participants and from an anchor whose correlation with MoCA change fell below the threshold the investigators had pre-specified. Three thresholds are therefore reported together, without adjustment for multiplicity, and none should be read as a validated responder definition. The analysis describes a shift in the distribution of change scores at the group level and does not establish that any individual participant experienced a clinically important benefit.

Supplementary Table S16. Missing-data pattern for the three co-primary outcomes and sensitivity of the primary estimate to the imputation procedure

Outcome	Timepoint	Observed	Missing	Missing (%)	Missing, intervention / control
**MoCA**	Baseline	355	0	0·0	0 / 0
**MoCA**	6 months	278	77	21·7	38 / 39
**SMI**	Baseline	354	1	0·3	1 / 0
**SMI**	6 months	278	77	21·7	38 / 39
**Trail Making Test A**	Baseline	254	101	28·5	50 / 51
**Trail Making Test A**	6 months	273	82	23·1	43 / 39
**Trail Making Test B**	Baseline	253	102	28·7	52 / 50
**Trail Making Test B**	6 months	272	83	23·4	43 / 40

All 355 randomised participants are included. The three registered co-primary outcomes are shown; the Trail Making Test contributes two measures, TMT-A and TMT-B. The 6-month clinical outcomes were missing for the same 77 participants (21·7%), balanced between groups, so the missing-data pattern for these outcomes is monotone. The Trail Making Test is an exception: baseline data were missing for more participants than 6-month data (19 participants had a 6-month but no baseline assessment), reflecting the baseline administration problem described in the Methods, so the pattern for that outcome is non-monotone.

Sensitivity of the primary MoCA estimate to the handling of missing data. The primary intention-to-treat analysis, which used 50 imputations, gave an adjusted between-group difference of 0·58 points (95% CI 0·03 to 1·13; p=0·039); the fraction of missing information was 0·26. A complete-case analysis of the same model in the full analysis set, which uses no imputed values, gave 0·61 points (0·10 to 1·13; p=0·020). Because the only incomplete clinical outcome was the 6-month assessment and the imputation model contained only the variables of the analysis model, multiple imputation and complete-case analysis estimate the same quantity here, and their close agreement indicates that the result does not depend on the imputation procedure. The estimate nonetheless remains close to the conventional significance threshold under both specifications.

Supplementary Table S17. System Usability Scale for the Fitbit device, by group

Measure	Intervention group	Control group
**System Usability Scale, mean (SD)**	57·00 (11·56)	55·44 (12·78)
**Participants completing the scale**	140	137
**Nearest adjective rating**	"OK"	"OK"

The System Usability Scale refers to the Fitbit device and was administered at the 6-month assessment. Participants in both groups wore the device during the assessment windows, so both were able to rate it. It was not a registered outcome and is reported as an exploratory measure of acceptability. The adjective rating follows the scale of Bangor et al.[SR7], in which "OK" corresponded to a mean score of 50·9; "OK" is the adjective nearest to the scores observed here. Scores were similar in the two groups; the trial was not designed to compare them and no formal comparison was made.

Supplementary Table S18. Tests of effect modification and within-subgroup treatment effects on MoCA

Analysis	n (intervention / control)	Estimate	95% CI	p value
**Tests of effect modification**				
**Treatment × suspected MCI**	278	0·05	−0·99 to 1·10	0·92
**Treatment × ApoE4 carriership**	278	0·60	−0·88 to 2·08	0·43
**Treatment × suspected MCI × ApoE4**	278	1·51	−1·55 to 4·56	0·33
**Within-subgroup adjusted differences**				
**Suspected MCI (MoCA <26)**	71 / 63	0·66	−0·27 to 1·58	0·16
**No suspected MCI (MoCA**	69 / 75	0·47	−0·10 to 1·03	0·10

**≥26)**				
**ApoE4 carrier**	21 / 19	0·97	−0·44 to 2·38	0·17
**ApoE4 non-carrier**	119 / 119	0·53	−0·04 to 1·11	0·068

All analyses are complete-case ANCOVAs of post-intervention MoCA in the full analysis set, adjusted for baseline MoCA and the covariates of the primary analysis. Estimates are in MoCA points.

The interaction terms test whether the treatment effect differs across subgroups; none provides evidence of effect modification. The within-subgroup differences are estimates of the treatment effect inside each subgroup and must not be read as evidence that the effect differs between subgroups, because the corresponding interaction tests are not significant. No adjustment for multiplicity has been made and the subgroups were defined on baseline characteristics only.

Supplementary Table S19. Derivation of the Fitbit- and Asken-derived measures and the valid-day criteria

Measure	Source field	Definition used	Valid-day criterion
**Daily step count**	Fitbit Web API, daily activity summary (steps)	Total steps recorded for the calendar day	At least 100 steps
**Total active time**	Fitbit Web API, daily activity summary (fairly and very active minutes)	Fairly active minutes plus twice the very active minutes; lightly active minutes not included	Recorded activity durations totalling 1152–1440 min (80–100% of the day)
**Total sleep time**	Fitbit Web API, sleep log (minutes asleep)	Minutes recorded as asleep, excluding time awake within the sleep period	As above
**Dietary nutrient intake**	Asken application (Asken Inc., Tokyo, Japan)	Nutrient totals computed by the application; entries made by manual input or by photograph with automatic recognition	Days with a completed record

The wear time of the device could not be retrieved directly for this study, so it was approximated by the total of the activity durations recorded for the day. The 80% threshold is taken from the WearTime80 criterion of Baroudi et al.[SR8], who compared definitions of a valid day in Fitbit data and found that the definition used can change population-level mean daily step count by up to about 2000 steps. Those authors required more than 80% of the day; the criterion applied here is 1152 min, which is 80% exactly, so days at the boundary are included here and excluded by their definition. Those authors also applied a criterion of more than 1000 steps per day; we used 100 steps because this population was older and less active, and a 1000-step criterion would have excluded genuinely low-activity days rather than non-wear days. Those authors defined wear time as the number of minutes with a registered heart rate divided by the total minutes in the day; the quantity used here is an approximation of that, obtained from the recorded activity durations rather than from the heart-rate record itself. The algorithms by which Fitbit classifies activity intensity and sleep are proprietary and have not been

published, so these measures are specific to the device and are not interchangeable with research-grade accelerometry or polysomnography. For the dietary records, only nutrient totals were available: food-level records were not exported, so intake cannot be reported by food group, and it cannot be determined whether a given record was entered manually or by photograph. Asken states that it calculates nutrient values from the Japanese Food Composition Table 2020 (eighth revised edition)[SR9,SR10]*, in which the method of calculating energy differs from that of earlier editions.*

Supplementary Table S20. Automated prompts sent to the intervention group

Prompt type	Participants sent this prompt	Prompts per participant, mean (SD)
**Weekly activity prompts**	146	16·9 (0·67)
**Weekly sleep prompts**	146	16·9 (0·75)
**Monthly activity prompts**	146	4·9 (0·38)
**Monthly sleep prompts**	146	4·9 (0·18)
**Monthly nutrition prompts**	118	3·5 (0·98)

Counts are taken from the prompt-delivery logs of the notification system. Activity prompts and sleep prompts were issued separately on the weekly and on the monthly schedule, so a participant could be sent all four. Nutrition prompts were issued monthly and only to participants who took up the optional dietary component, which is why fewer participants were sent them. Prompts were sent as SMS messages; the logs record issue and delivery by the messaging service, not receipt or reading by the participant, so these counts are an upper bound on exposure.

Supplementary Table S21. The three registered co-primary outcomes analysed jointly, with adjustment for multiplicity

Analysis set	Outcome	Adjusted difference (95% CI)	Unadjusted p	Holm-adjusted p, three outcomes	Holm-adjusted p, four outcomes
**ITT (primary)**	MoCA, points	0·58 (0·03 to 1·13)	0·039	0·117	0·156
	TMT-A, s	0·16 (−5·07 to 5·40)	0·95	—	1·0
	TMT-B, s	5·59 (−3·88 to 15·05)	0·25	0·50	0·75
	SMI, kg/m2	−0·01 (−0·09 to 0·07)	0·88	0·88	1·0
**FAS**	MoCA, points	0·61 (0·10 to 1·13)	0·020	0·060	0·080
	TMT-A, s	−0·72 (−6·17 to 4·72)	0·79	—	1·0

	TMT-B, s	3·27 (−6·73 to 13·27)	0·52	1·0	1·0
	SMI, kg/m2	−0·00 (−0·09 to 0·08)	0·92	1·0	1·0
**PP**	MoCA, points	0·92 (0·37 to 1·48)	0·001	0·003	0·004
	TMT-A, s	−1·04 (−7·36 to 5·29)	0·75	—	1·0
	TMT-B, s	1·97 (−9·65 to 13·60)	0·74	1·0	1·0
	SMI, kg/m2	0·00 (−0·11 to 0·11)	1·0	1·0	1·0

The trial registration and the protocol specified three primary outcomes — the Trail Making Test, the Montreal Cognitive Assessment, and the skeletal muscle mass index — with no ranking among them and no strategy for controlling multiplicity across them. This table applies the Holm procedure to those three outcomes as a single family, which is the analysis the registered plan would have required had a multiplicity strategy been specified. The registration does not state which part of the Trail Making Test represents that outcome, so two families are shown: one in which TMT-B represents the Trail Making Test, and one in which TMT-A and TMT-B are treated as separate endpoints. Substituting TMT-A for TMT-B in the three-outcome family leaves the adjusted value for MoCA unchanged. Estimates and unadjusted p values are those reported in tables S5 and S6.

In the intention-to-treat analysis, on which the primary inference rests, the adjusted p value for MoCA is 0·117 under the three-outcome family and 0·156 under the four-outcome family; under either, the difference is not statistically significant. Bonferroni adjustment gives the same value of 0·117. The adjusted value reaches conventional significance only in the per-protocol population, which is a post-randomisation subset reported as a sensitivity analysis. The analyses in the main text are reported unadjusted because no multiplicity-control strategy was pre-specified and introducing one after the fact would itself be a post-hoc decision; this table is provided so that the result under the registered outcome structure can be read directly.

Supplementary Table S22. Distribution of baseline MoCA scores

Baseline MoCA score	Participants, n (%)	Cumulative %
**≤19**	12 (3·4%)	3·4%
**20–21**	29 (8·2%)	11·5%
**22–23**	63 (17·7%)	29·3%
**24–25**	79 (22·3%)	51·5%
**26–27**	92 (25·9%)	77·5%
**28–29**	69 (19·4%)	96·9%
**30 (maximum score)**	11 (3·1%)	100·0%

Baseline MoCA was recorded at the screening assessment, which preceded randomisation. The mean was 25·1 (SD 3·0), the median 25, and the observed range 13 to 30. Of the 355 participants, 11 (3·1%) scored the maximum of 30 and 43 (12·1%) scored 29 or above, so the distribution was not compressed against the ceiling of the scale and there was room for improvement across most of the range. In all, 183 participants (51·5%) scored below 26, the cut-off validated for mild cognitive impairment in older Japanese.[20] Cognitive performance was not among the eligibility criteria, which were based on body-mass index, frailty status, and sarcopenia-related indicators. The level observed here is nonetheless higher than published values for Japanese community-dwelling older adults of similar age: a normative study of 1977 adults aged 65 years or older (mean age 73·6 years) reported a mean MoCA of 21·8 (SD 3·9), with 82·6% of scores below 26,[SR11] and a population-based cohort in Tokyo (496 adults, mean age 74·0 years) reported a mean of 23·7 (SD 3·6).[SR12] The expectation that cognitively unimpaired older adults score 27 or above derives from the North American sample in which the MoCA was developed; performance on the MoCA is sensitive to age, education, and the language of administration, and the Japanese data indicate that the standard cut-off of 26 falls above the average score of the general older Japanese population.

Supplementary Table S23. Fitbit-derived measures in natural units, by group and period

Measure	Group	Estimated mean before (95% CI)	Estimated mean after (95% CI)	Change	Difference in change
**Daily step count, steps**	Intervention	8269 (7357 to 9294)	8180 (7272 to 9201)	−89	+696
	Control	8715 (7777 to 9767)	7930 (7065 to 8900)	−786	—
**Total active time, min/day**	Intervention	68·1 (58·0 to 79·9)	75·3 (64·0 to 88·5)	+7·2	+6·6
	Control	75·7 (64·8 to 88·5)	76·4 (65·1 to 89·6)	+0·6	—
**Total sleep time, min/night**	Intervention	407·6 (391·1 to 424·7)	400·0 (383·8 to 417·0)	−7·6	+11·6
	Control	411·2 (395·0 to 428·1)	392·1 (376·4 to 408·4)	−19·1	—

Values are estimated marginal means from the generalised linear mixed models reported in table S7, back-transformed to natural units, for the per-protocol population (n=210; 114 intervention, 96 control). The models were fitted on the first 28 days of the wear period, before any prompt was sent, and the final 28 days, during which prompting was still being delivered. Change is the difference between the estimated means after and before within a group; difference in change is the corresponding contrast between groups. Confidence intervals are given for the estimated means; the inferential test is the group-by-period interaction reported as a ratio of

ratios in table S7, and no confidence interval is available for the natural-unit contrast. Because the models use a log link, these estimated means are slightly lower than the arithmetic means of the same data.

The direction of the effect matters for interpretation and is not the same for the three measures. For daily step count and total sleep time both groups declined over the 6 months and the intervention group declined less, so the between-group difference reflects a smaller decline rather than an increase. For total active time the intervention group increased while the control group was essentially unchanged, so that difference does reflect an increase. Baseline levels overlapped between the groups, although the estimated baseline active time was lower in the intervention group than in the control group.

Supplementary Figure

Section / topic	No	CONSORT 2025 checklist item	Reported in manuscript
**Title and abstract**			
**Title and abstract**	1a	Identification as a randomised trial	Title
**Title and abstract**	1b	Structured summary of the trial design, methods, results, and conclusions	Summary (Background, Methods, Findings, Interpretation, Funding)
**Open science**			
**Trial registration**	2	Name of trial registry, identifying number (with URL) and date of registration	Title block (Trial registration: UMIN-CTR, UMIN000052707; ClinicalTrials.gov, NCT06135740) and Methods — Study design and participants. Prospectively registered with UMIN-CTR on 6 Nov 2023 (publicly released 7 Nov 2023; record status "pre-initiation"), before the first participant was enrolled on 8 Nov 2023; additionally registered with ClinicalTrials.gov (named primary outcomes recorded 30 Nov 2023, as stated in the manuscript). Supplied here for completeness and not repeated in the manuscript: the UMIN record was publicly released on 7 Nov 2023 and the ClinicalTrials.gov record was first posted on 18 Nov 2023, that is after enrolment began; the manuscript therefore describes only the UMIN-CTR registration as prospective.
**Protocol and statistical analysis plan**	3	Where the trial protocol and statistical analysis plan can be accessed	Submitted alongside this manuscript as Supplementary Protocol and Supplementary Statistical Analysis Plan
**Data sharing**	4	Where and how the individual de-identified participant data (including data dictionary), statistical code and any other materials can be accessed	Data sharing statement in the manuscript. De-identified individual participant data will be considered for sharing with academic researchers on submission of a methodologically sound proposal, from 12 months to 5 years after publication, subject to institutional approvals, ethical constraints, and a signed data-access agreement; proposals should be directed to the corresponding author. The trial protocol and the statistical analysis plan are submitted with this manuscript. The data dictionary and the statistical code are not covered by the sharing statement.
**Funding and conflicts of interest**	5a	Sources of funding and other support (e.g., supply of drugs), and role of funders in the design, conduct, analysis and reporting of the trial	Methods — Role of the funding source; Declaration of interests; Contributors. The funding organisations had no role in the conduct of the trial, in data collection, or in the statistical analysis, and no access to individual-level data. Employees of NH Foods Ltd (protein-balanced food) and Google LLC (Fitbit devices) are co-authors and contributed, in that capacity, to the conception and design of the trial and to the review of the manuscript. Mitsui Fudosan managed the recruitment membership list and provided assessment space.
**Funding and conflicts of interest**	5b	Financial and other conflicts of interest of the manuscript authors	Declaration of interests
**Introduction**			
**Background and rationale**	6	Scientific background and rationale	Introduction
**Objectives**	7	Specific objectives related to benefits and harms	Introduction (final paragraph — hypothesis statement); Methods — Outcomes; Methods — Procedures (adverse-event monitoring)
**Methods**			
**Patient and public involvement**	8	Details of patient or public involvement in the design, conduct and reporting of the trial	Methods — Study design and participants. Patients and members of the public were not formally involved in the design, conduct, or analysis of the trial. Individual outcome results were returned to each participant by post, and overall findings will be disseminated to the cohort.
**Trial design**	9	Description of trial design including type of trial (e.g., parallel group, crossover), allocation ratio, and framework (e.g., superiority, equivalence, non-inferiority, exploratory)	Methods — Study design and participants. Prospective, single-centre, open-label, parallel-group randomised controlled trial with 1:1 allocation. The framework is a superiority comparison against an assessment-only control; no non-inferiority or equivalence margin was specified. The superiority framework is inferred from the design and was not stated in the registration or the protocol.
**Changes to trial protocol**	10	Important changes to the trial after it commenced including any outcomes or analyses that were not prespecified, with reason	Methods — Procedures: the baseline TMT was initially administered by all examiners under the same non-standard procedure and was re-administered under the standard procedure at the group orientation session; TMT is reported in full (Supplementary table S6) but interpreted as non-confirmatory. Methods — Outcomes: all three co-primary outcomes were registered without a hierarchy and are reported in full. Methods — Statistics: all three were analysed by the same ANCOVA, and MoCA was the only one to show a difference, so the exploratory analyses were all carried out on it, a choice made after the primary results and documented as a deviation from the registered outcome plan (Supplementary table S10).
**Trial setting**	11	Settings (e.g., community, hospital) and locations (e.g., countries, sites) where the trial was conducted	Methods — Study design and participants (community setting in Kashiwa, Japan; participants recruited by post from the membership register of the A-Shi-Ta community health-promotion facility, many of whose members also belong to the Kashiwa Study cohort in the same area)
**Eligibility criteria**	12a	Eligibility criteria for participants	Methods — Study design and participants (inclusion and exclusion criteria stated in text, including exclusion of current or previous users of a wearable device for self-management of health); Supplementary Table S1
**Eligibility criteria**	12b	If applicable, eligibility criteria for sites and for individuals delivering the interventions (e.g., surgeons, physiotherapists)	N/A — single-centre trial; no site- or deliverer-level eligibility criteria apply
**Intervention and comparator**	13	Intervention and comparator with sufficient details to allow replication. If relevant, where additional materials describing the intervention and comparator (e.g., intervention manual) can be accessed	Methods — Procedures (prompt targets stated in text: 3000/6000/8000 steps per day; 45/90/150 min exercise time per week; 5 and 6 h sleep with a 6–9 h maintenance band; 13%/15% of energy from protein with a 15–20% maintenance band); Supplementary Tables S2–S4
**Outcomes**	14	Prespecified primary and secondary outcomes, including the specific measurement variable, analysis metric, method of aggregation, and time point for each outcome	Methods — Outcomes (MoCA, TMT, SMI as primary; Fitbit-derived steps and total sleep time as secondary; frailty index and protein intake as other; assessment at 0 and 6 months)
**Harms**	15	How harms were defined and assessed (e.g., systematically, non-systematically)	Methods — Procedures (collected by spontaneous report plus systematic enquiry at scheduled outcome-assessment visits); Supplementary Methods, Method 4 (definitions, including the ICH-GCP definition for serious adverse events)
**Sample size**	16a	How sample size was determined, including all assumptions supporting the sample size calculation	Methods — Statistics (d = 0·34, two-sided α = 0·05, 80% marginal power per registered primary outcome, 20% attrition, 280 ÷ 0·80 = 350 randomised; natural-unit equivalents and the external standard deviations used are given in Supplementary table S1b). Multiplicity across the three co-primary outcomes was not incorporated.
**Sample size**	16b	Explanation of any interim analyses and stopping guidelines	N/A — no interim analyses or formal stopping rules were planned for this 6-month behavioural trial
**Randomisation: sequence generation**	17a	Who generated the random allocation sequence and the method used	Methods — Randomisation and masking (sequence generated in advance by the principal investigator from a random number table)
**Randomisation: sequence generation**	17b	Type of randomisation and details of any restriction (e.g., stratification, blocking and block size)	Methods — Randomisation and masking (permuted blocks of four, 1:1; no stratification)

Section / topic	No	CONSORT 2025 checklist item	Reported in manuscript
**Allocation concealment mechanism**	18	Mechanism used to implement the random allocation sequence, describing any steps to conceal the sequence until interventions were assigned	Methods — Randomisation and masking (allocation concealment was not implemented as a separate procedure: no envelope system or central allocation; the sequence was applied in a single pass after all enrolment and screening was complete); Supplementary Methods, Method 2 (what was and was not protected against, including that the assignment of the last participants in each block was predictable in principle); Discussion — dedicated paragraph on conduct-related bias
**Implementation**	19	Whether the personnel who enrolled and those who assigned participants to the interventions had access to the random allocation sequence	Methods — Randomisation and masking (the investigator who enrolled participants also applied the allocation sequence, had access to it, and was aware of the block size)
**Blinding**	20a	Who was blinded after assignment to interventions (e.g., participants, care providers, outcome assessors, data analysts)	Methods — Randomisation and masking (no one was masked; participants necessarily knew their group, and the staff who performed the 6-month assessments had also set up the devices, contacted participants, and managed the study, so they could often infer group assignment). Masking of the data analysts was not implemented and is not claimed.
**Blinding**	20b	If blinded, how blinding was achieved and description of the similarity of interventions	N/A — open-label trial; no blinding was implemented
**Statistical methods**	21a	Statistical methods used to compare groups for primary and secondary outcomes, including harms	Methods — Statistics (ANCOVA for clinical outcomes [MoCA, TMT, SMI, frailty, body composition]; GLMM for Fitbit-derived daily logs; ANCOVA for nutrition outcomes; harms summarised descriptively)
**Statistical methods**	21b	Definition of who is included in each analysis (e.g., all randomised participants), and in which group	Methods — Statistics. ITT = all 355 randomised participants. Full-analysis-set membership is outcome-specific and is defined by the presence of a post-baseline assessment: 140/138 for MoCA, 135/138 for TMT-A, and 135/137 for TMT-B. The corresponding complete-case ANCOVA samples, which additionally require a standard-procedure baseline value, are 128/126 for TMT-A and 126/126 for TMT-B (Supplementary table S6). PP = 114/96. All participants were analysed in the group to which they were assigned.
**Statistical methods**	21c	How missing data were handled in the analysis	Statistical analysis plan (multiple imputation with predictive mean matching; 50 imputations; Rubin's rules with the Barnard–Rubin small-sample degrees-of-freedom adjustment). Methods — Statistics states the method and the number of imputations and refers to the statistical analysis plan for the full specification.
**Statistical methods**	21d	Methods for any additional analyses (e.g., subgroup and sensitivity analyses), distinguishing prespecified from post hoc	Methods — Statistics. None of the additional analyses is documented as prespecified. The composition of the full-analysis-set and per-protocol populations, the treatment-by-adherence interaction, the exploratory subgroup analyses of ΔMoCA by baseline cognitive status and ApoE2/ApoE4 carrier status, the behaviour–cognition association analysis, and the modelling approaches for Fitbit-derived behaviour and Asken-derived nutrition were all specified during data analysis and manuscript preparation, as recorded in the statistical analysis plan. All are reported as post hoc and are uncorrected for multiplicity.
**Results**			
**Participant flow (with diagram)**	22a	For each group, the numbers of participants who were randomly assigned, received intended intervention, and were analysed for the primary outcome	Results and Methods — Study design and participants (2210 invited by post; 477 telephoned to arrange the explanation and screening visit; 48 declined at the explanation stage without proceeding to screening; 429 gave written informed consent and were screened; 74 did not meet the inclusion criteria; 355 randomised: 178 intervention and 177 control); Figure 1 (CONSORT flow diagram)
**Participant flow (with diagram)**	22b	For each group, losses and exclusions after randomisation, together with reasons	Figure 1 (CONSORT 2025 flow diagram)
**Recruitment**	23a	Dates defining the periods of recruitment and follow-up for outcomes of benefits and harms	Results (first paragraph: Nov 8, 2023 to Jul 31, 2024 for randomisation/primary completion; 6-month follow-up per participant)
**Recruitment**	23b	If relevant, why the trial ended or was stopped	N/A — trial reached its target enrolment and completed as planned; not stopped early
**Intervention and comparator delivery**	24a	Intervention and comparator as they were actually administered (e.g., who delivered, how participants adhered, whether they were delivered as intended (fidelity))	Methods — Procedures (algorithmic prompt delivery coupled to Fitbit data synchronisation; per-protocol intervention group wore Fitbit on a median of 121·8 days [IQR 87·3–145·5] of 180 days; human contact was limited to device set-up at a group orientation session and to technical support given in response to problems reported by participants; the registered plan for telephone follow-up of participants who did not change their behaviour or were not wearing the device was not implemented, and no participant was contacted because of their behavioural data)
**Intervention and comparator delivery**	24b	Concomitant care received during the trial for each group	Methods — Procedures (the control group received no prompts, advice, education, or other component of the intervention; participants in both groups remained free to use ordinary community and municipal health services, which were neither influenced nor recorded)
**Baseline data**	25	A table showing baseline demographic and clinical characteristics for each group	Table 1 (age, sex, education, BMI, ApoE2/ApoE4 carrier status, suspected MCI, MoCA, TMT-A, TMT-B, SMI, frailty index); Supplementary Table S12 by analysis population
**Numbers analysed, outcomes and estimation**	26	For each primary and secondary outcome, by group: number included in analysis; number with available data at the outcome time point; result for each group; estimated effect size and its precision (e.g., 95% CI); for binary outcomes, absolute and relative effect size	Results and Supplementary table S6 (adjusted between-group difference, 95% CI, p value, and numbers analysed for every outcome and analysis population). Observed group-specific means at baseline are given in Table 1; observed 6-month means by group are not tabulated for every outcome, and the corresponding estimates are reported as adjusted between-group differences.
**Harms**	27	All harms or unintended events in each group	Results — final paragraph before Discussion. No serious adverse events related to the wearable device, the nutrition application, or the protein-balanced food were reported in either group. Mild skin irritation at the Fitbit contact site was reported in both groups and stress related to dietary recording in the intervention group; these were captured by spontaneous report and by enquiry at the assessment visits, and participant-level counts by group were not systematically recorded. No participant withdrew because of an intervention-related adverse event.
**Ancillary analyses**	28	Any other analyses performed, including subgroup and sensitivity analyses, distinguishing pre-specified from post hoc	Results. None of these analyses is documented as prespecified: the composition of the full-analysis-set and per-protocol populations, the treatment-by-adherence interaction, the behaviour–cognition association analysis, and the exploratory subgroup analyses were specified during data analysis rather than in advance and are reported as post hoc exploratory analyses, uncorrected for multiplicity.
**Discussion**			
**Interpretation**	29	Interpretation consistent with results, balancing benefits and harms, and considering other relevant evidence	Discussion
**Limitations**	30	Trial limitations, addressing sources of potential bias, imprecision, generalisability, and, if relevant, multiplicity of analyses	Discussion — Limitations paragraphs (single-centre Japanese suburban setting; no masking; allocation concealment not implemented separately; 6-month duration; non-standard baseline TMT administration with re-administration and non-confirmatory interpretation; deviation from the registered outcome plan — three co-primary outcomes were registered without a hierarchy, all three were analysed identically, and the exploratory analyses were confined to MoCA because it was the only one to show a difference; possible measurement reactivity from asymmetric Fitbit wear; subgroup and exploratory analyses uncorrected for multiplicity)

Supplementary Fig.1: CONSORT 2025 checklist for the INSPIOR trial.

Completed checklist mapping each CONSORT 2025[17] item to its location in the main manuscript or in the supplementary materials. Items not applicable to this trial are marked "N/A" with the reason given.

Abbreviations: AE, adverse event; ANCOVA, analysis of covariance; ApoE, apolipoprotein E; BMI, body-mass index; CI, confidence interval; FAS, full analysis set; GLMM, generalised linear mixed model; ICH-GCP, International Council for Harmonisation – Good Clinical Practice; IQR, interquartile range; ITT, intention-to-treat; MCI, mild cognitive impairment; MICE, multiple imputation by chained equations; MoCA, Montreal Cognitive Assessment; PP, per protocol; RCT, randomised controlled trial; SMI, skeletal muscle mass index; TMT, Trail Making Test.

Section	CONSORT for Abstracts item	Reported in the abstract
**Title**	Identification of the study as randomised	Title: "… (INSPIOR): a single-centre, open-label, randomised controlled trial"
**Authors**	Contact details for the corresponding author	Title page (outside the abstract, as required by the journal)
**Trial design**	Description of the trial design (e.g., parallel, cluster, non-inferiority)	Methods: 6-month, single-centre, open-label, randomised controlled trial with two parallel groups (the 1:1 allocation ratio is given in the main text, not the abstract)
**Methods — Participants**	Eligibility criteria for participants and the settings where the data were collected	Methods: adults aged 65–92 with low body-mass index, pre-frailty, frailty, or sarcopenia-related decline, in Kashiwa, Japan
**Methods — Interventions**	Interventions intended for each group	Background describes the intervention (automated, wearable-based programme of activity, sleep, and optional nutrition prompts); Methods states the allocation to the Fitbit-based intervention or an assessment-only control
**Methods — Objective**	Specific objective or hypothesis	Background: whether the programme changed cognitive and physical function in at-risk older adults
**Methods — Outcome**	Clearly defined primary outcome for this report	Methods: three co-primary outcomes (MoCA, TMT, SMI), stated as having no pre-specified hierarchy and no multiplicity adjustment
**Methods — Randomisation**	How participants were allocated to interventions	Methods: allocated using permuted blocks of four; allocation was not concealed
**Methods — Blinding (masking)**	Whether participants, care givers, and those assessing the outcomes were masked to group assignment	Methods: allocation was not concealed and outcome assessors were not masked
**Results — Numbers randomised**	Number of participants randomised to each group	Findings: 355 randomised (178 intervention; 177 control)
**Results — Recruitment**	Trial status	Findings: Between Nov 8, 2023, and Jul 31, 2024 — the period covering randomisation and primary completion. The trial is complete; that status is stated in the main text rather than the abstract
**Results — Numbers analysed**	Number of participants analysed in each group	Findings: all randomised participants were analysed (intention-to-treat with multiple imputation)
**Results — Outcome**	For the primary outcome, a result for each group and the estimated effect size and its precision	Partially reported. Findings gives the adjusted between-group difference with its 95% CI and p value for each co-primary outcome (MoCA 0·58, 0·03 to 1·13, p=0·039; TMT-A 0·16 s, −5·07 to 5·40, p=0·95; TMT-B 5·59 s, −3·88 to 15·05, p=0·25; SMI −0·01 kg/m², −0·09 to 0·07, p=0·88). Results for each group separately are not given in the abstract, for length; they are reported in the main text and in Supplementary table S6.
**Results — Harms**	Important adverse events or side-effects	Findings: mild skin irritation and dietary-recording stress; no serious adverse event was intervention-related
**Conclusions**	General interpretation of the results	Interpretation: a small difference that did not survive multiplicity adjustment and is of uncertain clinical significance; no difference for the other two co-primary outcomes
**Trial registration**	Registration number and name of trial register	Methods: UMIN-CTR UMIN000052707; ClinicalTrials.gov NCT06135740
**Funding**	Source of funding	Funding: University of Tokyo–NH Foods Ltd Collaborative Research Programme; Google LLC; Mitsui Fudosan (in-kind support)

Supplementary Fig.2: CONSORT abstracts checklist for the INSPIOR trial.

Supplementary references

These references are cited only in this Appendix and are numbered independently of the reference list of the main manuscript.

[SR1] *Fukuoka Y, Narita T, Fujita H, et al. Importance of physical evaluation using skeletal muscle mass index and body fat percentage to prevent sarcopenia in elderly Japanese diabetes patients. J Diabetes Investig 2019; 10: 322–30.*

[SR2] *Hashimoto R, Meguro K, Lee E, Kasai M, Ishii H, Yamaguchi S. Effect of age and education on the Trail Making Test and determination of normative data for Japanese elderly people: the Tajiri Project. Psychiatry Clin Neurosci 2006; 60: 422–28.*

[SR3] *Nasreddine ZS, Phillips NA, Bédirian V, et al. The Montreal Cognitive Assessment, MoCA: a brief screening tool for mild cognitive impairment. J Am Geriatr Soc 2005; 53: 695–99.*

[SR4] *Kurata H, Meguro S, Abe Y, et al. Dietary protein intake and all-cause mortality: results from the Kawasaki Aging and Wellbeing Project. BMC Geriatr 2023; 23: 479.*

[SR5] *Masuoka N, Lei C, Li H, Hisatsune T. Influence of Imidazole-Dipeptides on Cognitive Status and Preservation in Elders: A Narrative Review. Nutrients 2021; 13: 397.*

[SR6]*Suzuki H. Instruction manual of the Japanese version of the Montreal Cognitive Assessment (MoCA-J), version 2.2. Fujiwara Y, supervising editor. Tokyo: Research Team for Social Participation and Community Health, Tokyo Metropolitan Institute of Gerontology, 2010.*

[SR7]*Bangor A, Kortum P, Miller J. Determining what individual SUS scores mean: adding an adjective rating scale. J Usability Stud 2009; 4: 114–23.*

[SR8]*Baroudi L, Zernicke RF, Tewari M, Carlozzi NE, Choi SW, Cain SM. Using wear time for the analysis of consumer-grade wearables' data: case study using Fitbit data. JMIR Mhealth Uhealth 2025; 13: e46149.*

[SR9]*Asken Inc. Basis of the nutrient calculation and of the judgement of excess and deficiency [in Japanese]. Tokyo: Asken Inc. https://www.asken.jp/info/347 (accessed Aug 4, 2026).*

[SR10]*Ministry of Education, Culture, Sports, Science and Technology, Japan. Standard tables of food composition in Japan — 2020 (eighth revised edition). Tokyo: Ministry of Education, Culture, Sports, Science and Technology, 2020.*

[SR11]*Narazaki K, Nofuji Y, Honda T, Matsuo E, Yonemoto K, Kumagai S. Normative data for the Montreal Cognitive Assessment in a Japanese community-dwelling older population. Neuroepidemiology 2013; 40: 23–29.*

[SR12]*Suzuki H, Kawai H, Hirano H, et al. One-year change in the Japanese version of the Montreal Cognitive Assessment performance and related predictors in community-dwelling older adults. J Am Geriatr Soc 2015; 63: 1874–79.*
