# Supplementary material for: Effect of a wearable-based individualised multidomain lifestyle intervention on cognitive and physical function in older adults at risk of decline (INSPIOR): a single-centre, open-label, randomised controlled trial
Source: eClinicalMedicine. 2026 Sep 23;100:104212. doi: 10.1016/j.eclinm.2026.104212 (PMC13634265; doi:10.1016/j.eclinm.2026.104212)
Supplement: Supplementary file 2 [file mmc2.pdf]

STUDY PROTOCOL

Impact of Notifications on Nutrition, Sleep, and Physical Activity on Intellectual Function and Muscle Mass in Older Adults (INSPIOR)

**Document type**	Trial protocol
**Document version**	1.0
**Document compiled**	5 January 2026
**Trial registration**	UMIN-CTR, UMIN000052707; ClinicalTrials.gov, NCT06135740
**First registered**	UMIN-CTR, 6 November 2023 (prospective, before first enrolment); ClinicalTrials.gov first posted 18 November 2023 (named primary outcome measures 30 November 2023)
**Ethics approval**	Ethics Committee of The University of Tokyo, approval no. 22-360
**Sponsor**	The University of Tokyo
**Principal Investigator**	Tatsuhiro Hisatsune, PhD
**Study site**	Graduate School of Frontier Sciences, The University of Tokyo, Kashiwa, Chiba, Japan
**Funding**	University of Tokyo–NH Foods Ltd Collaborative Research Programme; Google LLC; Mitsui Fudosan (recruitment logistics and assessment space)

Note on document timing and scope

This protocol document was compiled during preparation of the primary outcome manuscript to formalise, in narrative CONSORT/SPIRIT-style format, the trial design, eligibility criteria, intervention, and pre-specified outcomes of INSPIOR. The trial was prospectively registered with the UMIN Clinical Trials Registry (UMIN000052707) on 6 November 2023, before the first participant was enrolled on 8 November 2023. Five aspects of the registry record do not reflect the conduct of the trial. The masking field records "single (investigator)", whereas the trial was open-label (section 8). The control arm is registered as a sham comparator receiving regular care and health-related information, whereas no sham procedure was used and neither care nor health-related information was provided to that arm (section 5.2). The registered plan for telephone follow-up of participants who did not change their behaviour after an alert, or who were not wearing the device, was not implemented; human contact was limited to device set-up and to technical

support given in response to problems reported by participants (section 5.1). The registered intervention description refers to nutritional supplementation and promotion of exercise without indicating that the nutrition component, including the food provision, was optional and that its intake data came from a separate food-record application (section 5.1). The registry describes the trial as nested within an established cohort in Kashiwa City, whereas the sampling frame was the membership register of the A-Shi-Ta community health-promotion facility, many of whose members also belong to that cohort (section 4.1). A more detailed record was subsequently entered in ClinicalTrials.gov (NCT06135740), first submitted 13 November 2023 and first posted 18 November 2023, with the named primary outcome measures recorded on 30 November 2023. Apart from the five discrepancies set out above, the pre-specified outcomes, design, eligibility criteria, and intervention described in this document correspond to those entered into the trial registries; methodological details that go beyond the registry records (for example, statistical covariates, multiple-imputation specifications, and exploratory analysis families) are documented in the companion Statistical Analysis Plan and in the Methods section of the manuscript.

1. Background and rationale

Preserving cognitive function and delaying dementia are urgent public-health priorities as populations age. Multidomain interventions that simultaneously target physical activity, nutrition, cognitive engagement, and cardiovascular risk have shown small but inconsistent benefits on cognitive outcomes across landmark randomised trials and in systematic reviews of those trials. Most of these programmes, however, rely on intensive face-to-face coaching or human-delivered support, which limits their scalability to community settings.

Consumer wearable devices reliably increase daily step counts, and mobile-health interventions in older adults are usable, acceptable, and modestly effective at promoting physical activity. Higher daily step counts, greater volumes of structured exercise, and adequate sleep duration have each been associated with reduced dementia incidence in epidemiological cohorts and meta-analyses. To our knowledge, no completed randomised trial has tested a wearable-based multidomain intervention in which the behavioural content is generated and delivered automatically from objective daily lifelog data, with a cognitive outcome among the primary outcomes.

INSPIOR will test whether a 6-month, algorithm-driven, wearable-based multidomain intervention combining personalised prompts for physical activity and sleep with an optional nutrition component can improve cognition and physical function in community-dwelling older adults at increased risk of physical decline.

2. Objectives and hypotheses

2.1 Primary objective

To evaluate the effect of a 6-month, wearable-based, algorithm-driven multidomain intervention on cognitive function and muscle mass in community-dwelling older adults at increased risk of physical decline, compared with an assessment-only control.

2.2 Hypothesis

Compared with an assessment-only control, the multidomain intervention will improve global cognition, executive function, and skeletal muscle mass at 6 months.

2.3 Secondary and exploratory objectives

Secondary objectives are to evaluate the effect of the intervention on objectively measured daily steps and total sleep time. Other pre-specified objectives are to evaluate the effect of the intervention on frailty index and dietary protein intake. Exploratory objectives, documented in the companion Statistical Analysis Plan and reported in the corresponding manuscript, include additional Fitbit-derived behavioural measures, additional dietary outcomes, body-composition measures, system usability, exploratory subgroup effects defined by baseline cognitive status and ApoE genotype, and exploratory associations between behavioural change and change in cognition.

3. Trial design

INSPIOR is a prospective, single-site, open-label, parallel-group randomised controlled trial with 1:1 allocation and a 6-month primary endpoint. Recruitment is from the membership register of the A-Shi-Ta community health-promotion facility in Kashiwa City, Chiba, Japan, whose members are community-dwelling older adults living independently. Many of these members also belong to the established Kashiwa Study cohort in the same area. The primary purpose, as registered, is prevention. The experimental arm is the wearable-based multidomain intervention; the comparator is an assessment-only control arm, which received no prompts, advice, or other component of the intervention. It is not a sham-controlled trial, and the comparator is not an active comparator.

4. Eligibility criteria

Eligibility criteria are as registered at ClinicalTrials.gov (NCT06135740).

4.1 Inclusion criteria

Participants must meet all of the following:

- Aged 65 years or older (recruited up to 92 years).
- Living in Japan.
- Living independently with or without carer support, and not requiring nursing care provided by long-term care insurance.
- Able and willing to wear a Fitbit device for ≥20 hours per day, including during sleep, for the study duration.
- Able and willing to receive and act on interventions.
- Has capacity to provide written informed consent.
- Smartphone access to allow Fitbit installation and setup.

- Has at least one of the following risk indicators: BMI <20 kg/m² for ages 65–69 years or <21.5 kg/m² for ages ≥70 years; pre-frailty (FRAIL questionnaire score 1–2) or frailty (FRAIL score ≥3); sarcopenia-related decline indicated by low grip strength (female <18 kg, male <28 kg), gait speed <1.0 m/s, or low skeletal muscle mass index (female <5.7 kg/m², male <7.0 kg/m²).

4.2 Exclusion criteria

- Receiving full-time care in a care facility.
- Unable to receive or act on study interventions.
- Existing wearable users, or prior experience using a wearable to manage their health.
- Uncontrolled hypertension, uncontrolled diabetes, or uncontrolled chronic kidney disease, or presence of cardiac pacemakers or cardiovascular stents.
- Any additional condition or situation that the Principal Investigator considers inappropriate for participation.
- Absence of any of the risk indicators specified in the inclusion criteria.

5. Intervention

5.1 Intervention group

Participants assigned to the intervention group receive a Fitbit Charge 5 device (Google LLC, Mountain View, CA, USA) and are asked to wear it continuously for 6 months, including during sleep. The first 28 days are a baseline measurement window in which no prompt is sent; prompting begins one month after wear starts and continues for the remaining 5 months. Daily lifelog data are processed by an algorithm to generate automated, personalised SMS prompts on physical activity (steps, exercise time) and sleep (duration and sleep–wake variability), targeted to each participant's recent behavioural state. Prompts are generated on both monthly and weekly schedules and are delivered in Japanese.

Participants may opt in to an additional nutrition component, in which dietary intake is logged via the Asken food-management smartphone application (Asken Inc., Tokyo, Japan), and automated prompts are generated to support adequate protein intake. To reduce the financial burden of increasing protein intake, opt-in participants may also receive a protein-balanced food (NH Foods Ltd, Tokyo, Japan) free of charge.

5.2 Control group

Participants assigned to the control group receive no prompts, advice, or other component of the intervention. Control participants wear a Fitbit and record diet via Asken during the baseline and end-of-study assessment windows only, for pre–post comparison, and receive no intervention prompts based on these records at any point.

6. Outcomes

Outcomes are as registered at ClinicalTrials.gov (NCT06135740), with the primary outcome measures registered on 30 November 2023. All outcomes are assessed at baseline (0 months) and at 6 months.

6.1 Primary outcomes

- Executive function — Trail Making Test (TMT), assessed at 0 and 6 months.
- General cognition — Montreal Cognitive Assessment (MoCA), assessed at 0 and 6 months.
- Muscle mass — skeletal muscle mass index (SMI), assessed at 0 and 6 months.

6.2 Secondary outcomes

- Amount of exercise — step counts measured by Fitbit, assessed at 0 and 6 months.
- Amount of sleep — total sleep time measured by Fitbit, assessed at 0 and 6 months.

6.3 Other pre-specified outcomes

- Frailty/sarcopenia — frailty index, assessed at 0 and 6 months.
- Malnutrition — protein intake, assessed at 0 and 6 months.

6.4 Exploratory outcomes (not registered as pre-specified outcomes)

In addition to the registered outcomes above, the following exploratory measures are collected and analysed under the companion Statistical Analysis Plan: additional Fitbit-derived behavioural measures (e.g. total active time); additional Asken-derived dietary outcomes (energy, lipid, carbohydrate, dietary fibre); body-composition measures (muscle mass, body weight, body-fat percentage, BMI); the System Usability Scale (SUS) for the wearable device; and exploratory subgroup analyses of change in MoCA defined by baseline cognitive status (suspected mild cognitive impairment defined as MoCA <26) and ApoE2/ApoE4 carrier status. These exploratory outcomes are identified as exploratory in the corresponding manuscript and are interpreted as hypothesis-generating.

7. Sample size

Power was calculated for the registered primary outcomes, assuming a standardised effect size of $d = 0.34$, a two-sided α of 0.05, and 80% power, with 140 evaluable participants per group. Using standard deviations from external reference populations, $d = 0.34$ corresponds to 0.27 kg/m² for SMI, 9.4 s for TMT-A, 31.3 s for TMT-B, and 1.1 points for MoCA. Allowing for 20% attrition, 280 evaluable participants require 350 randomised participants (280 ÷ 0.80). The 80% figure is the marginal power for each outcome considered alone; multiplicity across the three co-primary outcomes, and the joint power of demonstrating an effect on all three, were not incorporated into the sample size. Recruitment continued until the target was reached.

8. Randomisation and masking

The allocation sequence is generated in advance by the Principal Investigator from a random number table, using permuted blocks of four in a 1:1 ratio. Eligibility assessment, informed consent, and the screening examination — which also serves as the baseline outcome assessment — are completed before any allocation is applied; the sequence is then applied in a single pass, assigning participants in the fixed order in which they were screened. The one exception is the baseline Trail Making Test, which had to be re-

administered after allocation for the reason described in the corresponding manuscript. Allocation concealment was not implemented as a separate procedure; this is acknowledged as a limitation in the corresponding manuscript. No one was masked after allocation and the trial is open-label. Participants necessarily knew their group, and the staff who performed the 6-month assessments had also set up the devices, contacted participants during the intervention, and managed the study, so they could often infer group assignment; at a single site of this size these roles could not be separated. The masking field of the ClinicalTrials.gov record (NCT06135740) states “single (investigator)”, which does not reflect the conduct described here.

9. Statistical analysis (overview)

The detailed statistical methods are specified in the companion Statistical Analysis Plan. Three outcomes (TMT, MoCA, SMI) were registered as primary, with no ranking among them and no strategy for controlling multiplicity across them. All three were analysed in the same way. The analysis that the corresponding manuscript reports in most detail is an intention-to-treat (ITT) ANCOVA of post-intervention MoCA, with treatment group as the main effect and pre-specified covariates (baseline MoCA, age, sex, BMI, years of education, ApoE2 carrier status, ApoE4 carrier status). Missing post-baseline outcomes are handled by multiple imputation, with estimates pooled using Rubin's rules. Sensitivity analyses are conducted in the full-analysis-set and per-protocol populations. The same ANCOVA framework is applied to the other clinical outcomes (TMT, SMI, frailty index, body-composition measures). Fitbit-derived daily logs are analysed in the per-protocol population using generalised linear mixed models. Behaviour–cognition associations, nutrition outcomes, and subgroup effects are analysed as exploratory analyses.

10. Data collection and management

Baseline demographic and health information, cognitive assessments, body-composition measurements, and questionnaire data are collected by trained study staff at the study site. Fitbit data are synchronised through the standard Fitbit cloud service and exported to the study database. Dietary data are exported from the Asken application. Source documents and electronic records are retained for the duration required by institutional policy and applicable law. Access to the raw individual-participant dataset is restricted to the academic study team at The University of Tokyo.

11. Adverse events

Adverse events are defined as any untoward medical occurrence reported by participants during the trial, including device-related skin irritation, sleep disturbance attributed to prompt notifications, and psychological distress associated with dietary recording. Events are collected through spontaneous report and through systematic enquiry at scheduled outcome-assessment visits. Serious adverse events are defined according to ICH-GCP criteria and reported to the Ethics Committee of The University of Tokyo.

12. Ethics, consent, and dissemination

The trial was approved by the Ethics Committee of The University of Tokyo (approval no. 22-360) and is conducted in accordance with the Declaration of Helsinki and applicable national regulations. Written informed consent is obtained from all participants prior to enrolment. The trial is registered with the UMIN Clinical Trials Registry (UMIN000052707) and ClinicalTrials.gov (NCT06135740). Individual outcome results are returned to each participant by post. Overall findings are disseminated through peer-reviewed publication, a symposium hosted by The University of Tokyo, and the laboratory website. Reporting follows CONSORT 2025 guidelines for parallel-group randomised trials.

13. Data sharing

Deidentified individual participant data will be considered for sharing with academic researchers on submission of a methodologically sound proposal, beginning 12 months after publication and ending 5 years after publication, subject to institutional approvals, ethical constraints, and a signed data-access agreement. Proposals should be directed to the corresponding author.

14. Funding and role of the funding source

Funding is provided by the University of Tokyo–NH Foods Ltd Collaborative Research Programme and by Google LLC. NH Foods Ltd supplies the protein-balanced food. Google LLC provides the Fitbit devices and a research donation. Mitsui Fudosan supports recruitment logistics and provides some assessment space. The study sponsor (The University of Tokyo) has final responsibility for the study. The funding organisations have no access to individual-level study data and no role in the conduct of the trial, in data collection, in the statistical analysis, or in the decision to submit the manuscript for publication. Employees of NH Foods Ltd and Google LLC are named investigators and, in that capacity, contributed to the conception and design of the trial and to the review of the manuscript; their contributions are listed in the Contributors section of the manuscript. The corresponding author has full access to all study data and final responsibility for the decision to submit.
