# Supplementary material for: Effect of a wearable-based individualised multidomain lifestyle intervention on cognitive and physical function in older adults at risk of decline (INSPIOR): a single-centre, open-label, randomised controlled trial
Source: eClinicalMedicine. 2026 Sep 23;100:104212. doi: 10.1016/j.eclinm.2026.104212 (PMC13634265; doi:10.1016/j.eclinm.2026.104212)
Supplement: Supplementary file 3 [file mmc3.pdf]

STATISTICAL ANALYSIS PLAN

Impact of Notifications on Nutrition, Sleep, and Physical Activity on Intellectual Function and Muscle Mass in Older Adults (INSPIOR)

**Document type**	Statistical Analysis Plan
**Document version**	1.0
**Document compiled**	5 January 2026
**Trial registration**	UMIN-CTR, UMIN000052707; ClinicalTrials.gov, NCT06135740
**Outcomes registered**	30 November 2023 (primary, secondary, and other pre-specified outcomes)
**Related protocol**	INSPIOR Trial Protocol v1.0, compiled 5 January 2026
**Sponsor**	The University of Tokyo
**Principal Investigator**	Tatsuhiro Hisatsune, PhD

Note on document timing and scope

This Statistical Analysis Plan (SAP) documents the statistical methods of the INSPIOR randomised controlled trial. The trial was prospectively registered with the UMIN Clinical Trials Registry (UMIN000052707) on 6 November 2023, before the first participant was enrolled. The trial's primary, secondary, and other pre-specified outcomes — listed in Section 3.1 — were subsequently recorded at ClinicalTrials.gov on 30 November 2023, prior to substantive accrual of outcome data; no statistical methodology was registered alongside the outcomes.

The methodological choices specified in this SAP fall into two categories. The following elements were established by the study team prior to outcome analysis, although not formally registered: the covariate set for the primary ANCOVA (baseline outcome value, age, sex, BMI, years of education, ApoE2 and ApoE4 carrier status).

The following analyses were specified during data analysis and manuscript preparation, are documented in this SAP for transparency, and are reported as exploratory in the corresponding manuscript: the treatment-by-adherence interaction analysis; the specific modelling approach for Fitbit-derived daily behaviour; the specific modelling approach for Asken-derived nutrition outcomes; the behaviour–cognition association analysis; and the exploratory subgroup analyses of ΔMoCA defined by baseline cognitive status and ApoE2/ApoE4 carrier status.

Deviations between the registered outcome plan and the present manuscript — in particular the cautious, non-confirmatory treatment of TMT owing to the use of a non-standard baseline administration procedure that led to re-administration of TMT in a group setting (see below) — are described explicitly in the Methods and Discussion sections of the corresponding manuscript.

1. Introduction

INSPIOR is a 6-month, single-site, open-label, parallel-group randomised controlled trial with 1:1 allocation, comparing a wearable-based, algorithm-driven multidomain intervention with an assessment-only control in community-dwelling older adults aged 65–92 years at increased risk of cognitive or physical decline. This SAP describes the statistical methods applied to evaluate the effect of the intervention.

2. Trial design and objectives

The primary objective is to evaluate the effect of the intervention on cognitive function and muscle mass at 6 months, with corresponding secondary objectives for objectively measured daily steps and total sleep time, and other pre-specified objectives for frailty index and dietary protein intake. The trial design and eligibility criteria are summarised in the companion protocol.

3. Outcomes

3.1 Outcomes pre-specified at ClinicalTrials.gov registration (30 November 2023)

The following outcomes were registered at ClinicalTrials.gov as the trial's pre-specified outcomes and are assessed at baseline (0 months) and at 6 months.

Primary outcomes:

- Executive function — Trail Making Test (TMT).
- General cognition — Montreal Cognitive Assessment (MoCA); range 0–30.
- Muscle mass — skeletal muscle mass index (SMI), kg/m^2.

Secondary outcomes:

- Daily step counts (Fitbit-derived).
- Total sleep time (Fitbit-derived).

Other pre-specified outcomes:

- Frailty index.
- Dietary protein intake.

3.2 Additional outcomes analysed under this SAP (not registered as pre-specified)

In addition to the registered outcomes above, the following exploratory outcomes are analysed under this SAP. They are identified as exploratory in the corresponding manuscript and interpreted as hypothesis-

generating.

- Additional Fitbit-derived behavioural measures, including total active time.
- Additional Asken-derived dietary measures: energy, lipid, carbohydrate, and dietary fibre intake.
- Body-composition measures: muscle mass, body weight, body-fat percentage, BMI.
- System Usability Scale (SUS) for the wearable device.
- Exploratory subgroup analyses of ⊿MoCA defined by baseline cognitive status (suspected mild cognitive impairment, MoCA <26) and ApoE2/ApoE4 carrier status.
- Exploratory associations between 6-month behaviour change and ⊿MoCA.

4. Sample size

Assuming a standardised effect size of d = 0.34, a two-sided α of 0.05, and 80% power for each registered primary outcome, 280 evaluable participants (140 per group) are required; allowing for 20% attrition, this corresponds to 350 randomised participants (280 ÷ 0.80).

5. Analysis populations

Three analysis populations are defined for the present manuscript. The specific compositions of these populations were determined during data analysis and manuscript preparation.

Intention-to-treat (ITT). All randomised participants, analysed in the group to which they were assigned. This is the primary analysis population for the registered clinical outcomes, with missing post-baseline outcomes handled by multiple imputation.

Full analysis set (FAS). All randomised participants with a post-baseline outcome assessment for the outcome of interest, irrespective of completeness of Fitbit or Asken recording. Used for sensitivity analyses and complete-case comparisons.

Per-protocol (PP). Participants who completed the data-collection protocol assigned to their group. For the intervention group, this requires Fitbit use over the 6-month period together with agreement to record diet in the dietary application, during the last five months of which algorithm-driven prompts targeting activity and sleep were delivered on the basis of the Fitbit records, together with prompts on protein intake based on the dietary records. For the control group, this requires Fitbit and dietary data during the baseline and end-of-study assessment windows used for pre–post comparison. Participants who declined dietary recording are excluded from this population; agreeing to record does not guarantee usable records in the analysis windows. The PP population is used for sensitivity analyses, behavioural (Fitbit) analyses, nutrition analyses, and behaviour–cognition association analyses.

6. Primary analysis

Three outcomes (TMT, MoCA, SMI) were registered as primary, with no ranking among them. All three were analysed in the same way. The analysis that the corresponding manuscript reports in most detail is an

ANCOVA fitted to the post-intervention (6-month) MoCA in the ITT population, with treatment group as the main effect and the following covariates: baseline MoCA, age, sex, BMI, years of education, ApoE2 carrier status, and ApoE4 carrier status. The estimand for each of the three co-primary outcomes is the adjusted between-group difference in its mean value at 6 months. Education and ApoE carrier status were included as established correlates of cognition; their relationship with skeletal muscle mass is not well established, and for SMI and the body-composition outcomes they were retained for uniformity. The treatment effect is reported as the adjusted mean difference with two-sided 95% confidence interval and p value.

The same ANCOVA framework is applied separately to TMT and SMI as outcomes registered alongside MoCA. As described in the corresponding manuscript, TMT is reported in full but is not treated as a confirmatory outcome because its baseline administration initially followed a non-standard procedure and TMT was re-administered under the standard procedure in a group setting, leaving baseline TMT available for fewer participants and under different conditions than the other outcomes; this is documented as a deviation from the registered outcome plan.

7. Handling of missing data

Missing post-baseline clinical outcomes (MoCA, TMT, SMI, frailty index, body-composition measures) are imputed using multiple imputation in R with predictive mean matching for continuous variables. The imputation model is the same as the analysis model: it includes treatment group, the baseline value of the outcome, and the covariates listed in Section 6, and no other variables. Baseline TMT is itself missing for 101 (TMT-A) and 102 (TMT-B) participants for the reason described in the corresponding manuscript. These participants are retained in the intention-to-treat analyses of TMT: the baseline TMT term is applied only to participants for whom a standard-procedure baseline score exists, and the remaining covariates are used for those for whom it does not. Baseline TMT is not itself imputed.

Estimates from the $m = 50$ imputed datasets are pooled using Rubin's rules with the Barnard–Rubin small-sample degrees-of-freedom adjustment.

8. Sensitivity analyses

8.1 Sensitivity analyses across populations

Sensitivity analyses of the MoCA effect are conducted as complete-case ANCOVAs in the FAS and the PP populations, with the same covariate set as the primary analysis. Results across the ITT, FAS, and PP populations are presented as a forest plot.

8.2 Treatment-by-adherence interaction

To formally evaluate whether the treatment effect on MoCA is larger in adherent participants, a complete-case ANCOVA is fitted in the FAS with a treatment-by-adherence interaction term, where adherence is coded 1 for PP participants and 0 for FAS-only participants. Covariates match the primary analysis. The interaction coefficient and its 95% CI are reported. As adherence is a post-randomisation variable that may

itself reflect prognostic factors, this analysis is exploratory.

9. Other clinical outcomes

SMI, frailty index, and the body-composition measures (muscle mass, body weight, body-fat percentage, BMI) are analysed using the same ANCOVA framework as MoCA, with the corresponding baseline value of the outcome included as a covariate alongside the covariates listed in Section 6. ITT estimates use multiple imputation with Rubin's rules; FAS and PP estimates are complete-case ANCOVAs. Adjusted mean differences with 95% CIs and p values are reported.

10. Fitbit-derived daily behaviour

Fitbit-derived daily logs (daily steps, total active time, total sleep time) are analysed in the PP population using generalised linear mixed models (GLMMs) with random intercepts at the participant level and fixed effects for treatment group, period (pre vs post), and their interaction. Pre and post periods are defined as the first 28 days of the 6-month wear period, which precede the start of prompting, and the final 28 days, during which prompting is still being delivered. The treatment-by-period interaction is reported on the ratio-of-ratios scale; values >1.0 indicate a relative increase in the intervention group versus control.

11. Exploratory nutrition outcomes

Exploratory nutrition outcomes (protein, lipid, carbohydrate, dietary fibre, and total energy intake) are derived from the Asken food-management application. For each participant, the mean daily intake is computed over two 14-day windows: weeks 3–4, immediately before prompting begins, and weeks 24–25, near the end of the wear period and after dietary prompting had ended — a narrower window than for Fitbit logs because of higher daily participant burden, and a within-window mean rather than a daily GLMM because of day-level missingness.

Means from the follow-up window are analysed by ANCOVA in the PP population, with the corresponding pre-intervention mean and the same covariates as the primary MoCA analysis. Adjusted between-group differences with 95% CIs and p values are reported.

12. Behaviour–cognition association analysis

Exploratory associations between 6-month behaviour change and ΔMoCA are assessed in the PP population using a single ANCOVA model with post-intervention MoCA as outcome and three behaviour-change predictors entered simultaneously: change in exercise (composite z-score of change in daily steps and change in total active time), change in sleep (change in total sleep time), and change in protein intake. The model also adjusts for baseline MoCA, the corresponding pre-intervention value of each behaviour-change predictor, and the same covariates as the primary MoCA analysis. Missing post-baseline behaviour data are handled with the same MI specification as the primary analysis. Standardised regression coefficients are reported.

13. Subgroup analyses

Exploratory subgroup analyses of the intervention effect on ⊿MoCA are conducted in the FAS so that observed ⊿MoCA can be used without imputation. Subgroups are defined by baseline suspected MCI status (MoCA <26 vs ≥26), ApoE4 carrier status, ApoE2 carrier status, and the intersection of suspected MCI and ApoE4 status. Given small subgroup sizes, between-group comparisons of ⊿MoCA use two-sided Mann–Whitney U tests; rank-based effect sizes (*r*), approximate 95% CIs based on the observed SD and per-arm n, and unadjusted p values are reported. These analyses are exploratory and uncorrected for multiplicity.

14. Adverse events

Adverse events are summarised descriptively by treatment group, with categorisation by relatedness (device-related, app-related, food-related, other) and seriousness. Participant-level counts were not systematically recorded, so counts and percentages of participants reporting each event are not reported. No formal between-group statistical testing is pre-specified.

15. General reporting conventions

Continuous variables are summarised as mean (SD). Categorical variables are summarised as n (%). Effect estimates are reported with two-sided 95% confidence intervals. p values are reported to two significant figures. All tests are two-sided unless explicitly stated otherwise. A significance threshold of $\alpha = 0.05$ is used for each of the three co-primary outcomes considered separately; no adjustment for multiplicity across them was pre-specified.

16. Software

All analyses are conducted in R. Multiple imputation of the clinical outcomes uses the mice package with predictive mean matching for continuous variables; pooling uses Rubin's rules with the Barnard–Rubin small-sample degrees-of-freedom adjustment. ANCOVA models use the lm() function; GLMMs use the lme4 package.

17. Deviation from the registered outcome plan

Deviations between the registered outcome plan and the present manuscript are described explicitly in the Methods and Discussion sections of the corresponding manuscript. The principal deviation concerns the TMT. TMT was prespecified as a primary outcome in the registered protocol. However, no official training programme or authorised Japanese-language script exists for TMT, and all examiners initially administered it under the same non-standard procedure, in which the examiner does not point out sequencing errors and records the number of errors instead; under the standard procedure the examiner corrects errors in real time while timing continues, so that the completion time includes the time taken to correct them. The discrepancy was identified by a co-investigator who was not one of the examiners, at a meeting held after all baseline assessments had been completed but before the intervention began. TMT was re-administered under the

standard procedure at the group orientation and device-setup session, which took place after allocation and was the first day of the intervention and therefore the last opportunity for a pre-intervention measurement; the group format and the limited time available meant that not all participants could be re-assessed. As a result, baseline TMT data were available for fewer participants (254 of 355 for TMT-A and 253 of 355 for TMT-B; 255 had at least one valid score and 252 had both) and were obtained at a different time and in a different setting from the other baseline outcomes, whereas the 6-month TMT was administered under the standard procedure concurrently with the other outcomes. TMT is therefore reported in full in the present manuscript (Supplementary table S6) using the same ANCOVA framework, but — because the analysis conditions on the less reliable baseline scores — is interpreted as non-confirmatory rather than excluded. This issue was specific to the administration of TMT and did not affect the administration or scoring of MoCA, SMI, or the body-composition outcomes.
